# The landscape of public-private partnerships in global health governance: introducing a new dataset

**DOI:** 10.1186/s12992-025-01162-z

**Published:** 2025-11-24

**Authors:** Leah Shipton

**Affiliations:** School of Public Policy, Simon Fraser University, 515 West Hasting Street HC3265, Vancouver, BC V6B 5K3 Canada

**Keywords:** Public-private partnerships, Global health governance, Multistakeholder governance, Decision-making power, Inequity, Global health

## Abstract

**Background:**

Global health public-private partnerships are prominent actors and forums for the governance of global health. They channel significant funding into global health and shape policy priorities and options for pressing health problems. Led by state and non-state actors, they are often championed as inclusive governing spaces. Despite their prominence, there is no up-to-date, comprehensive analysis of the quantity and qualities of global health public-private partnerships, including the distribution of decision-making power among their governing board members.

**Results:**

This article analyzes a new dataset of 73 global health public-private partnerships governed by a total of 630 actors. These analyses offer three high-level insights. First, high-income country representatives hold 69% of seats on partnership governing boards. Thus, while public-private partnerships have expanded the types of actors that can participate in governance, there remain significant disparities in access to decision-making based on country income-level. Second, a typology of public-private partnerships based on the composition of decision-makers on governing boards is presented. The typology includes Business, Civil Society, Trio, and Super public-private partnerships, of which Trio and Civil Society partnerships are the most common. Third, as public-private partnerships themselves hold governing seats in 24 partnerships, this article lends support to the idea that some partnerships are gaining agency and autonomy in global health through inter-partnership cooperation. Additional analyses shed light on the timeline of the rise of public-private partnerships and a range of characteristics, including their headquarter location, function, health issues addressed, and legal status.

**Conclusions:**

This article provides a big picture perspective on key patterns in the characteristics and distribution of decision-making power of global health public-private partnerships. Together, the analyses suggest that moving from multilateral governance through international organizations like the World Health Organization, to multistakeholder governance through public-private partnerships has contributed to a decrease in decision-making influence for low and middle-income countries and an increase for high-income countries. In doing so, it lays the groundwork for scholarly and practitioner debate about the appropriate distribution of decision-making power in global health governance.

**Supplementary Information:**

The online version contains supplementary material available at 10.1186/s12992-025-01162-z.

## Background

Public-private partnerships (PPPs) have become a common feature of global health governance, like global governance more broadly. Their prominence reflects a decades-long move toward multistakeholderism in global governance, where state *and* non-state actors work together to address global issues [1]. Global multistakeholderism may take the form of ancillary engagement, where multilateral organizations (e.g., World Health Organization) consult with non-state actors on their various governing tasks (e.g., policy design or implementation) [2]. For example, the United Nations Framework Convention on Climate Change is the central state-based institution for global climate governance, but non-state actors can participate as observers during Conferences of Parties to shape its implementation [3]. Likewise, the United Nations Food Systems Summit engages non-state actors in dialogue around policy, partnership building, and financing [4]. This article explores the executive form of global multistakeholderism, where decision-making itself is multistakeholder, rather than multilateral [2]. Global health PPPs can therefore be understood as a more institutionalized form of multistakeholderism.^1^

Despite the prominence of PPPs, there has not been a recent comprehensive analysis of the distribution of decision-making influence within and among active PPPs in global health. Buse and Harmer [5] offered one of the earliest accounts of representation on a sub-set of global health PPP, focusing on low and middle-income countries (LMICs), corporations, and non-governmental organizations. Westerwinter’s [6] extensive dataset on PPPs in global governance includes some analysis of the functions and institutional design of global health PPPs. It also traces the participation of states, international organizations, businesses, and non-governmental organizations in global health PPP governance, but without additional information on the country, region, or income-level that those stakeholders represent. Thus, gaps remain in understanding the range of constituencies governing global health PPPs and how decision-making influence or power – as reflected in the number and proportion of seats – is distributed among these constituencies. In other words, who governs global health PPPs? How many are there? And what are notable patterns and characteristics of PPPs in global health governance?

To answer these questions, I assemble and analyze a novel *Global Health Public-Private Partnership Dataset* of global health PPPs to illustrate the range and distribution of partnership types, characteristics, and governing constituencies. In the process, I make four contributions to the literature and debates on PPPs in global health governance. First and foremost, I find that while public sector representation on the governing boards of PPPs is reasonable, these representatives from high-income countries, the United States in particular,^2^ hold far more seats, and therefore decision-making influence, than their low, lower-middle, and upper-middle income country counterparts combined. This trend holds with non-state governors, who are predominantly from high-income countries. Indeed, my dataset reveals that high-income country representatives from all constituencies hold 69% of governing seats in global health PPPs. Thus, while global health PPPs broaden the range of actors that participate in global health governance, the dataset analyses suggest that in the move from multilateral venues like the World Health Organization (WHO) to PPPs, high-income countries have secured more decision-making power.

Second, recognizing the diversity and vagueness with which PPPs are often discussed in global health literature and discourse, I propose a typology that categorizes PPPs based on their board constituency composition. Third, I find that existing PPPs have governing seats on a third of PPPs, lending support for de Bengy Puyvallée’s [7] argument that a sub-set of global health PPPs have evolved beyond short-term programs into transnational bureaucracies with agency and authority of their own to shape global health governance. The presence of PPPs as half the governors of the Access to COVID-19 Tools Accelerator (ACT-A), a super PPP [8] that coordinated the global COVID-19 response exemplifies this trend.

Overall, this novel dataset and analyses provide a bird’s eye view of PPPs in global health governance, confirming troubling disparities in decision-making influence that contradict now mainstream decolonization and equity discourses in global health. Likewise, it opens opportunity for large-n research on global health PPPs, which are typically studied through small-n case studies [9–13]. These data and findings will be of use to scholars and practitioners interested in engaging PPPs that address different global health issues, confronting disparities in decision-making influence in global health, and debating what an appropriate distribution of decision-making influence would be within global health PPPs – just as has been done around gender representation in global health organizations and committees [14, 15] and LMIC representation in global health publishing and conferences [16–18].

In the sections that follow, I first present the study methods, including the PPP definition and inclusion criteria for the dataset, data collection process, and data preparation and analysis. Next, I present the findings of the descriptive analyses of the dataset and then conclude with a commentary on the most compelling outcomes and patterns from the findings.

## Methods

### PPP definition and dataset inclusion criteria

I define global health PPPs as collaborative arrangements that bring together at least one private sector (e.g., philanthropic foundation, corporation, non-governmental organization) and one public sector (e.g., state, international organization) actor to achieve a health promoting goal across multiple international jurisdictions [6, 19–21].^3^ While some scholars restrict PPPs to those involving at least one public sector and one business sector actor [20, 21], and others require them to involve at least one each of public, business, and civil society stakeholder [6], to capture the variety of PPPs in global health, I broadened the operating definition for this dataset simply to include at least one public and one private sector stakeholder.

In line with this definition, five criteria were used to determine if a PPP would be included in the dataset, as assessed by a review of each prospective PPP’s website, reports, governing documents, or other relevant materials. The first criterion was purpose; PPPs were included in the dataset if they had an explicit goal to promote health. This criterion is in place because health has many determinants, so PPPs addressing any number of issues, such as climate change or gender equality, could be included. However, while these topics are related to health, PPPs focusing primarily on these issues do not necessarily fit as actors in or venues for global health governance. Thus, if health was not mentioned as an explicit goal of a PPP, it was not included. Motivation for this criterion came from an observation of Westerwinter’s [6] dataset of transnational PPPs, which includes 163 PPPs labeled as health initiatives. However, upon closer inspection, many of these PPPs do not explicitly pursue a health promoting goal. For example, Westerwinter categorizes HeforShe, a global gender equality initiative, and Diamond Development Initiative International, a responsible artisanal mining partnership, as health partnerships. While gender equality and mining projects have clear implications for health, these PPPs do not have an explicit aim to address health issues and therefore would not have a meaningful role in global health governance.

The second criterion was that the PPP has a mandate and/or operations that span multiple countries. Thus, a PPP was excluded if its mandate and/or operations were limited to one country. This criterion is in place to ensure the dataset focuses on PPPs that are part of the global health governance architecture. While national or sub-national PPPs can have ties to global funding sources, they are generally understood as part of the public health infrastructure of one country. They do not typically influence governance processes at the global level. This criterion thus draws on Andonova’s (15, p213) definition of the *global* in global PPPs as referring to “partnerships that operate within the broad frameworks of the multilateral system and connect diverse sets of actors across jurisdictions in response to problems with global dimensions.”

The third and fourth criteria required PPPs to have a governing body and that governing body must have public and private sector membership. As one of the aims of this dataset is to map the distribution of decision-making influence within and across global health PPPs, they must have a governing venue such that PPP governors can be identified. I drew partially on Westerwinter’s [6] measure of forum to determine whether PPPs have a governing body that meets these two criteria. Thus, PPPs were included if they have a governing body with decision-making power to steer the direction of the PPP, and if they have a governing body with at least one public and one private sector member with substantial influence as governor. On the latter point, while data were collected on members with observer status on governing boards, they were not counted in the dataset analyses discussed below as they were determined as not having sufficient decision-making influence over the partnership. Relatedly, and consistent with principal-agent theory, which is commonly used to study PPPs in global governance [22], members of PPP secretariats with board seats (e.g., CEOs) were not counted in the dataset analyses. This is because ‘principals’ (i.e., non-secretariat PPP board members), who have ultimate authority over and responsibility for the PPP’s strategic, financial, and legal oversight, delegate authority to the ‘agent’ (i.e., PPP secretariat) to act on their behalf, including by implementing their decisions [22]. As such, the dataset focuses on the principals’ decision-making power in PPPs, as reflected in their seats on the governing board. The fifth criterion was that the PPP is active, as evidenced by an up-to-date website, reports or other documents, and/or professional social media profiles (e.g., LinkedIn).

### Data collection

I began data collection by compiling a list of possible PPPs to vet for inclusion in the dataset. I compiled this initial set of PPPs from existing research articles or datasets [6, 14, 23, 24]. None of these resources were sufficient on their own as the basis of the dataset for this research. Global Health 50/50 [14] and Hoffman & Cole [23] list a range of global health organizations, not just PPPs, so while they were a helpful way to identify 10–20 PPPs, they are not a comprehensive list to rely on for the entirety of the dataset. World Health Organization Maximizing Positive Synergies Collaborative Group [24], though a helpful resource for some PPPs, had many programmatic PPPs that did not fit the criterion of having a governing body or PPPs that have since ended. Westerwinter [6] has the largest dataset of PPPs. In addition to the issue raised above regarding its broad definition of health, I did not use this dataset verbatim for two reasons. First, while the dataset collects valuable information about the number of each type of actor governing the partnership, and the design and function of the partnership, it does not include sufficient data on governing members, which was crucial to my interest in the distribution of decision-making power in global health governance via PPPs. Second, upon further inspection of the dataset, I found many PPPs to be no longer functioning and several did not appear to fit the definition or inclusion criteria for PPPs. For example, Canada’s international development agency, the International Development Research Center, which is a Canadian federal Crown corporation, was included in the dataset as a PPP. For these reasons, I opted to create a new dataset.

Based on the above listed resources and my own knowledge of global health PPPs, between September 2023 and January 2024 I compiled an initial list of 265 potential PPPs to vet for inclusion in the dataset using the criteria described above. The vetting process took place in early 2024, leading to the inclusion of 73 PPPs in the dataset. In mid-2024, data were collected on three aspects of PPPs: scope, function(s), and decision-makers. For the scope of the PPPs, I collected information on the year they were founded, the health issue they address, headquarter country, region of operation, legal status, host organization (if applicable), and whether they portray themselves explicitly as a PPP (including through the language of multistakeholder partnership). For the next aspect, I used Westerwinter’s [6] categorization of PPP functions, though with some modifications (in brackets): knowledge creation and exchange, agenda setting (and advocacy), standard setting (and policymaking), funding (or financing), implementation (including partnership coordination), capacity building, service provision, and monitoring. I also added one more function common to global health PPPs: product development and market shaping.

The most extensive area of data collection was for information about PPP decision-makers. For each PPP, I collected data on the total number of governors, total number of governors per constituency type, total number of private, public, and existing PPP governors, the names, country, and region of governing individuals and organizations, and the names of individuals and organizations with observer status on the governing body (if applicable). Some of the constituency types were identified before data collection began, though the list expanded as needed during the data collection process. The final dataset included 11 constituency types. The first eight were categorized by World Bank classifications of low, lower-middle, upper-middle, and high-income country location: academic institutions, civil society organizations, community groups, companies, independent representatives (i.e., those who do not represent a sector or organization), philanthropic foundations, medical institutions (namely hospitals), and states. In this dataset, community groups are those with members who belong to an affected, often marginalized, group and have a mandate to represent and advance the needs of that group (e.g., people living with HIV, youth) [25]. This is distinct from civil society organizations, which may lend support to community groups but are not characterized by their staff belonging to those affected groups, nor do their organizational mandates typically focus on advancing the needs of a specific group. Philanthropic foundations are distinguished by their considerable financial assets, often provided by an individual donor or corporation, which are used for foundation activities or donated to other organizations or groups to promote a public good [26]. The remaining three constituencies were industry associations, international organizations, and existing PPPs. Data were not collected on the income-level of the countries where these organizations were hosted because they represent a broad set of stakeholders spanning multiple country income levels. For example, the WHO is headquartered in Switzerland, but it represents Member States of every income-level.

### Data preparation and analysis

The dataset was prepped for upload to RStudio for analysis in fall 2024. Four main descriptive analyses were conducted using the dataset. First, recognizing the diversity of PPPs within the dataset, the data were categorized into four types of PPPs, and a simple analysis was conducted to determine the proportion of each type of PPP within the full dataset. Second, I completed a simple time series analysis to track the cumulative rise of each global health PPP type over time. Third, I assessed partnership characteristics, including the range of PPP headquarter locations and proportion of PPPs based in each headquarter location, the legal status of PPPs, including how many were independent versus hosted, and what proportion of PPPs framed themselves as partnerships. This set of analyses also involved identifying the range and proportion of health issues addressed by and functions of PPPs in the dataset. Lastly, I conducted constituency analyses, which assessed the number and proportion of constituencies with governing seats in the PPPs by income-level and region (according to WHO region classifications). I completed these analyses for the entire global health PPP dataset.

## Results

Seventy-three global health PPPs were identified, appraised, and selected for inclusion in the *Global Health Public-Private Partnership Dataset* (see additional file 1 for the full list). Below, I present the results of the descriptive analyses in three sections: partnership typology and timeline, partnership characteristics, and dataset constituency analysis.

### Partnership typology and timeline

Global and public health officials and organizations use the term public-private or multistakeholder partnership in varied and vague ways [27]. Over the course of compiling the dataset, I came across organizations that refer to themselves as PPPs because their membership base has public and private stakeholders who participate in their activities and access their resources, even if their governance is not by public and private actors. Other organizations call themselves PPPs because they work with other partners to implement a program, but again, governance of the program is not hybrid. These varied understandings of PPPs were largely addressed in the vetting process when applying the PPP inclusion criteria. However, as referred to when defining PPPs earlier, the global health PPP literature also wrangles with different definitions of the term. This includes debating whether a PPP requires a for-profit private sector governing member or whether it should have at least one each of civil society, government, and business sector stakeholder on the governing body [6, 20, 21].

**Fig. 1:**
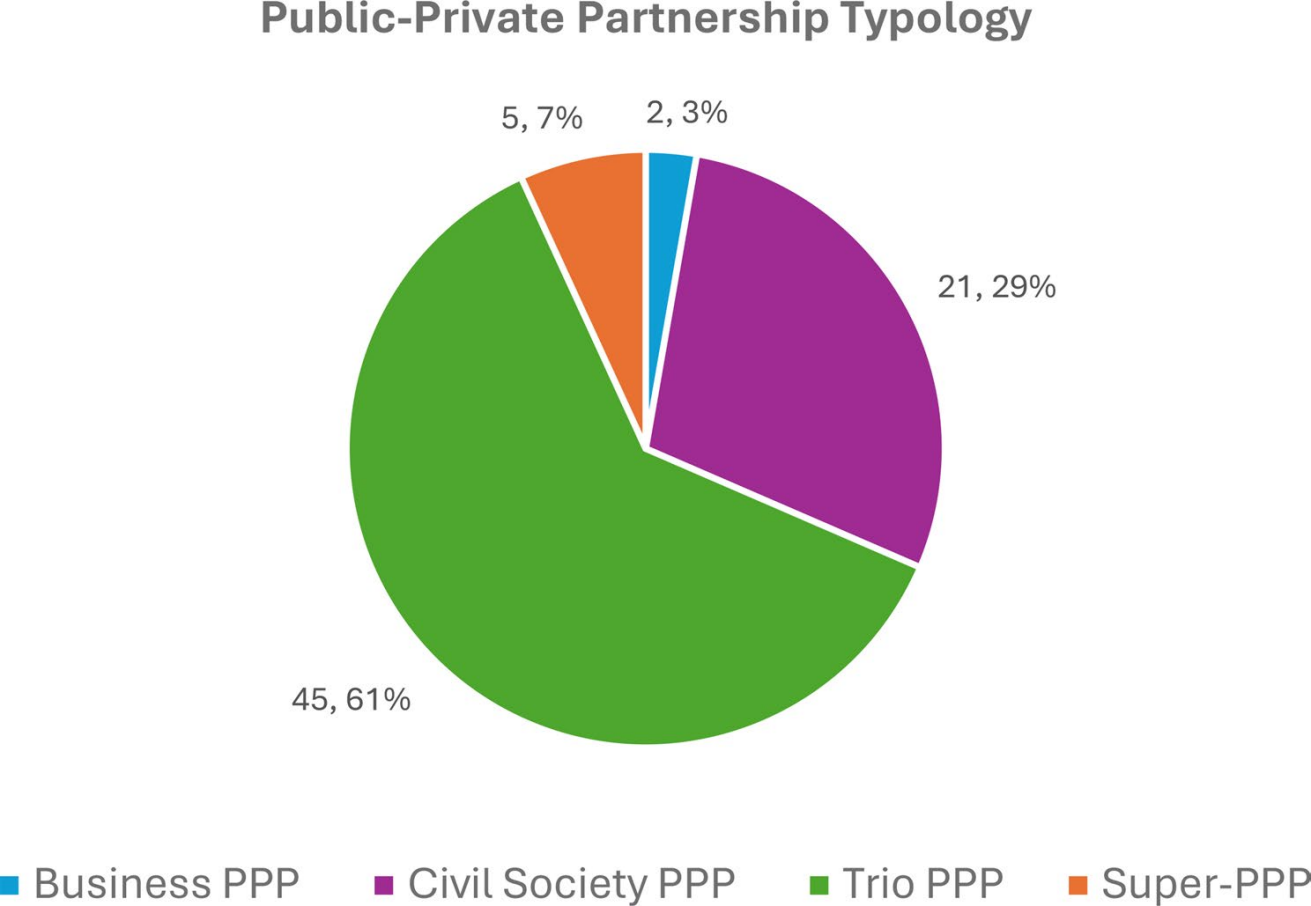
Proportion of global health PPP types

Rather than adjudicate between these definitions, the first contribution from my dataset is a typology of PPPs that acknowledges this diversity. Figure 1 presents the distribution of four partnership types across the PPP dataset: Business, Civil Society, Trio, and Super PPPs. Business PPPs are those whose governors include at least one public sector (state or international organization) and at least one for-profit company or industry association representative, but do not include other private sector representatives. My search found only two global health business PPPs in the dataset: the Innovative Health Initiative and the International Conference on Harmonization of Technical Requirements for Registration of Pharmaceuticals for Human Use. Next are civil society PPPs, which include at least one public sector representative and at least one not-for-profit private sector representative, but no for-profit representatives. The not-for-profit private sector is a broad category including representatives of civil society, community, philanthropic foundations, medical associations, and those who hold seats as independent board members. Global health civil society PPPs comprise 29% (21/73) of the PPP dataset and include prominent PPPs such as the Alliance for Health Policy and Systems Research, Drugs for Neglected Diseases Initiative, Global Financing Facility, Global Polio Eradication Initiative, Global Road Safety Facility, and Unitaid. By far, the most common partnership type are trio PPPs, which make up 61% (45/73) of PPPs in the dataset. These PPPs include at least one public sector, one for-profit private sector, and one not-for-profit private sector representative. Notable PPPs in this partnership type are the Coalition for Epidemic Preparedness Innovations, Foundation for Innovative New Diagnostics, Gavi, The Vaccine Alliance (Gavi), Global Fund to Fight AIDS, Tuberculosis, and Malaria (The Global Fund), Pandemic Fund, Scaling Up Nutrition, Stop TB Partnership, and UHC2030.

Lastly, 7% (5/73) of partnerships belong to the super PPP category. I define super PPPs as partnerships for which at least 20% of governors comprise existing PPPs of any type (i.e., business, civil society, or trio PPPs). I impose this threshold because, rather interestingly, and telling of the rising authority and agency of PPPs in global health governance via inter-partnership cooperation [7], existing PPPs hold seats in 24 of the 73 PPPs in the dataset. However, in most cases, PPPs are a small percentage of board member composition in PPPs. As my typology differentiates PPPs based on *who* (i.e., what types of stakeholders) has decision-making power in a PPP, as indicated by governor membership composition, I see it as important for super PPPs to be distinguished by having substantial representation of existing PPPs as governors. This is compatible with existing characterizations of super PPPs, which highlight that they bring together existing PPPs in a way that obscures public sector representation, accountability, and transparency due to multiple layers of governance that dilute the direct influence that any one public (and private) sector representative of one PPP can have on a particular governing task [28]. In other words, while a PPP board governor has direct (albeit, shared with co-governors) influence over decision-making for that PPP, that same governor has diluted influence over decision-making of a super PPP because in this model they are sharing decision-making with several other existing PPP governing boards, in addition to standalone public or private sector governors. To meet this distinguishing feature of diluted or obscured decision-making influence, super PPPs require a substantial portion of its governors to be existing PPPs.

To illustrate, while UHC2030 has one PPP (Gavi) as governor, the presence of eight private and 11 public sector governors means that, by and large, most governors have a direct line of influence over the UHC2030 partnership. By comparison, five out of 10 governors of the ACT-A are PPPs (or 50% of governors), which significantly obfuscates the influence that any one board member of those existing PPPs can have on the ACT-A’s direction. To be clear, the ACT-A is the most ideal example of a super PPP in terms of the dominance of existing PPP governor representation. Other super PPPs, in order of proportion of existing PPP governor representation, include the Global Oxygen Alliance (50%), Global Water Operators Partnership Alliance (31%), Food Fortification Initiative (20%), and Global Alliance for Vitamin A (20%).

**Fig. 2:**
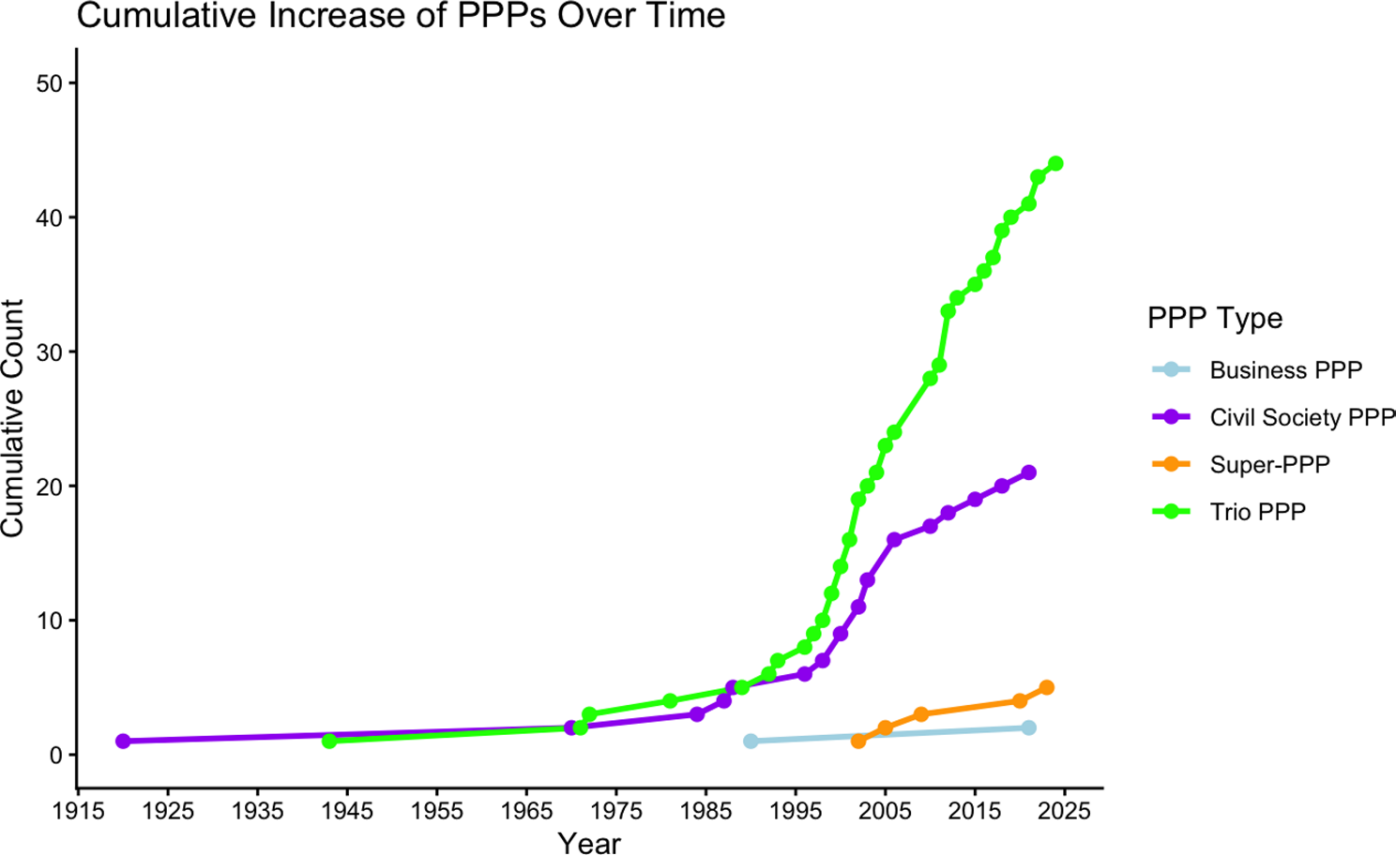
Increase of global health PPPs over time by type

Notably, as illustrated in Fig. 2, the last three super PPPs with lesser PPP representation preceded the COVID-19 pandemic. The super PPPs with the most PPP representation were created for the pandemic response (ACT-A) and as a continuation of medical oxygen governance after the pandemic (Global Oxygen Alliance). Like the three pre-COVID-19 super PPPs, the Health 8, another super PPP launched in 2007 but not included in the dataset as it is unclear if it is still operational, also had substantial minority PPP governing representation, with 25% of its governors (2/8) from the PPP constituency [29]. This suggests that super PPPs are not necessarily a partnership model created during the COVID-19 pandemic (and post-pandemic) era, as has been previously argued [8]. Besides the emergence of super PPPs, Fig. 2 shows an expedited increase of civil society and trio global health PPPs in the 2000s onwards, a trend that has previously been tied to the influx of philanthropic funds into global health [30].

### Partnership characteristics

*PPP headquarters*,* legal status*,* and partnership framing*. By far, Switzerland (42%) and the United States (24%) are the most common headquarter locations for global health PPPs in the dataset. The remaining PPPs are headquartered primarily in other European countries or Canada. Only 4% of PPPs have headquarters in African countries; two in Kenya and one in South Africa. Likewise for the Western Pacific Region where Japan, South Korea, and Taiwan each host one PPP. The Southeast Asia region only headquarters one PPP (in India) and the Eastern Mediterranean region hosts none. Indeed, altogether, Europe and North America headquarter 60% and 31% of global health PPPs, respectively. Additional file 2 summarizes the list of headquarter locations. Turning to their legal status, 63% of global health PPPs are hosted, with the remaining 37% of PPPs incorporated as non-profit charities or foundations. Of the 45 hosted PPPs, twenty-six (58%) of them are hosted by international organizations. This aligns with existing knowledge that international organizations are aware of their environment and other actors when they design their programs [31], and may in fact be the entrepreneurial force behind the rise of PPPs [19]. The WHO, for example, began pursuing and coordinating health collaborations with non-state actors, including through PPPs such as the Roll Back Malaria Partnership, shortly after The Global Fund’s launch in 2002 to protect and/or extend its influence and focality in global health [32–35]. The remaining 18 (40%) of PPPs are hosted by non-profit charities or foundations and one is hosted by a state (Swedish government agency). Among international organizations, the WHO serves as the host of eight global health PPPs plus two other PPPs for which it shares hosting responsibilities (Global Oxygen Alliance with UNICEF and Unitaid and UHC2030 with the Organisation for Economic Co-operation and Development and World Bank). The World Bank hosts five PPPs, the UN Office for Project Services hosts four, and the remaining PPPs are hosted by the Food and Agricultural Organization [2], UN Environmental Programme [2], UNICEF [2], United Nations Economic Commission for Europe [1], and UN Habitat [1].

Lastly, while the majority of PPPs (66%, 48/73) explicitly portray themselves as a public-private or multistakeholder partnership on their websites, social media profiles, or other organizational material, or explicitly state on their websites or legal documents that the PPP is governed by multistakeholder representatives, a substantial minority (34%, 25/73) of civil society and trio PPPs do not. Notable PPPs that avoid this framing yet are consistently referred to as PPPs in scholarship and practice [6, 14, 23, 24, 28, 36], include Foundation for Innovative New Diagnostics, Global Alliance for Nutrition, Nutrition International, and TB Alliance. Whether this is an intentional evasion of the terminology, as Lie [11] argues in the Scaling Up Nutrition Movement’s insistence on framing itself as a country-driven movement despite its increasingly formalized global public-private governance structure, in an attempt to legitimize itself to multiple audiences, is for future empirical inquiry.

**Fig. 3:**
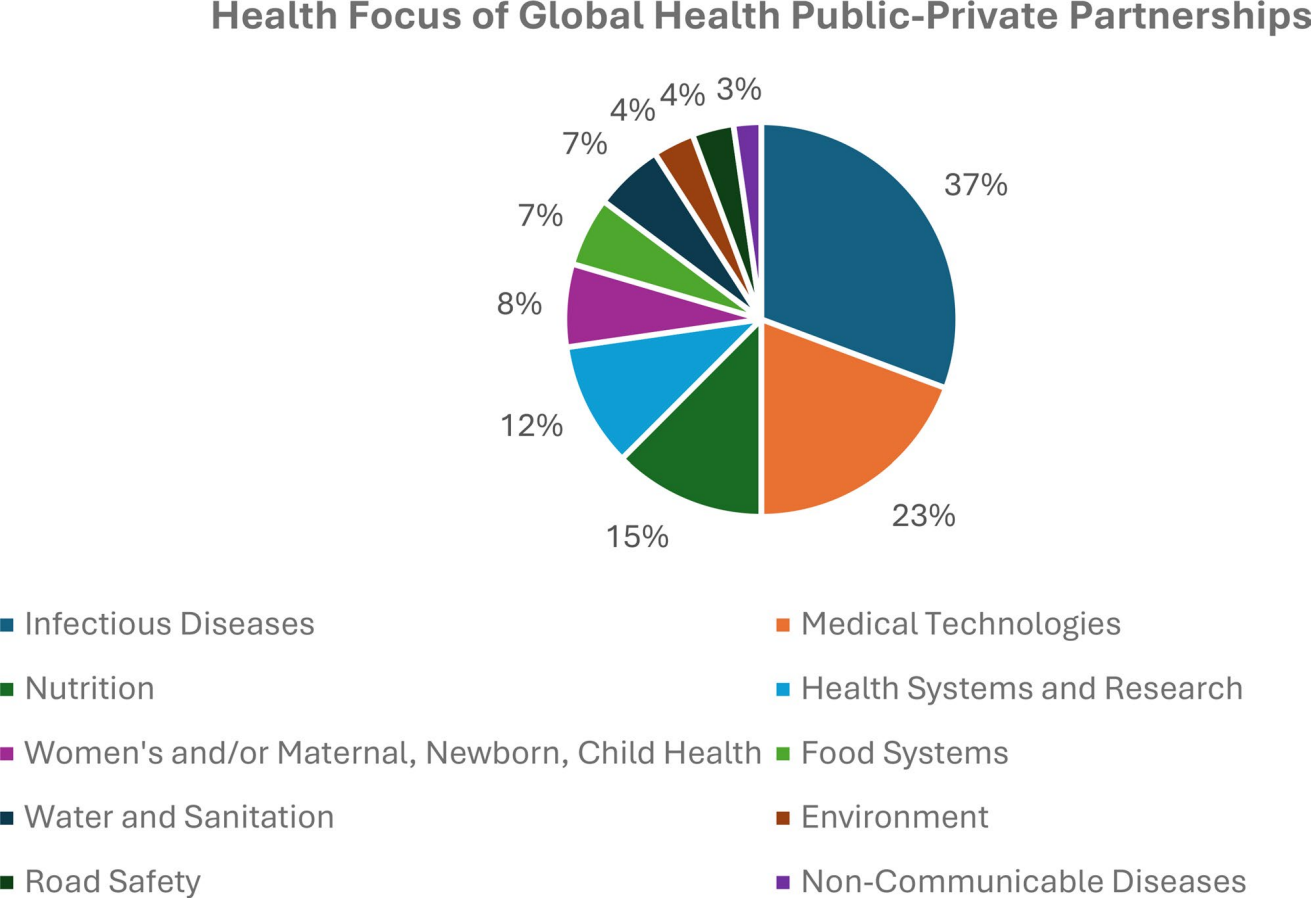
Health issues targeted by global health PPPs.^1^ Note that PPPs can address multiple health issues and so the data in the figure does not add up to 73 PPPs or 100%

*Health issues.* Predictably, the most common health issue targeted by global health PPPs in the dataset are infectious diseases (27/73, 37%), often addressed through medical technologies like vaccines, which the second largest number of PPPs focus on (17/73, 23%). Still, a solid number of PPPs address nutrition (11/73, 15%) and health systems and research (9/73, 12%). The latter encompasses a range of PPPs whose work falls broadly under the category of funding, conducting, or supporting health systems, policies, or research. This includes the Alliance for Health Policy and Systems Research, Guidelines International Network, Research4Life, UHC2030, among others. The three (4%) PPPs addressing environmental issues (other than water and food systems) include the Global Partnership on Plastic Pollution and Marine Litter, International Centre of Insect Physiology and Ecology, and Strategic Approach to International Chemicals Management. In line with global health funding flows [37], only 3% (2/73) of global health PPPs address non-communicable diseases. Granted, PPPs focused on nutrition, environmental issues, and health systems, policies, and research may impact non-communicable disease outcomes. Still, this lack of attention to non-communicable diseases seems out-of-touch, at least to some extent, with the priorities of many countries. Indicative, non-communicable diseases make up 35%, 53%, and 76% of the disease burden in low-income, lower-middle-income, and upper-middle-income countries, respectively [38]. Figure 3 depicts the issues addressed by PPPs in the dataset.

**Fig. 4:**
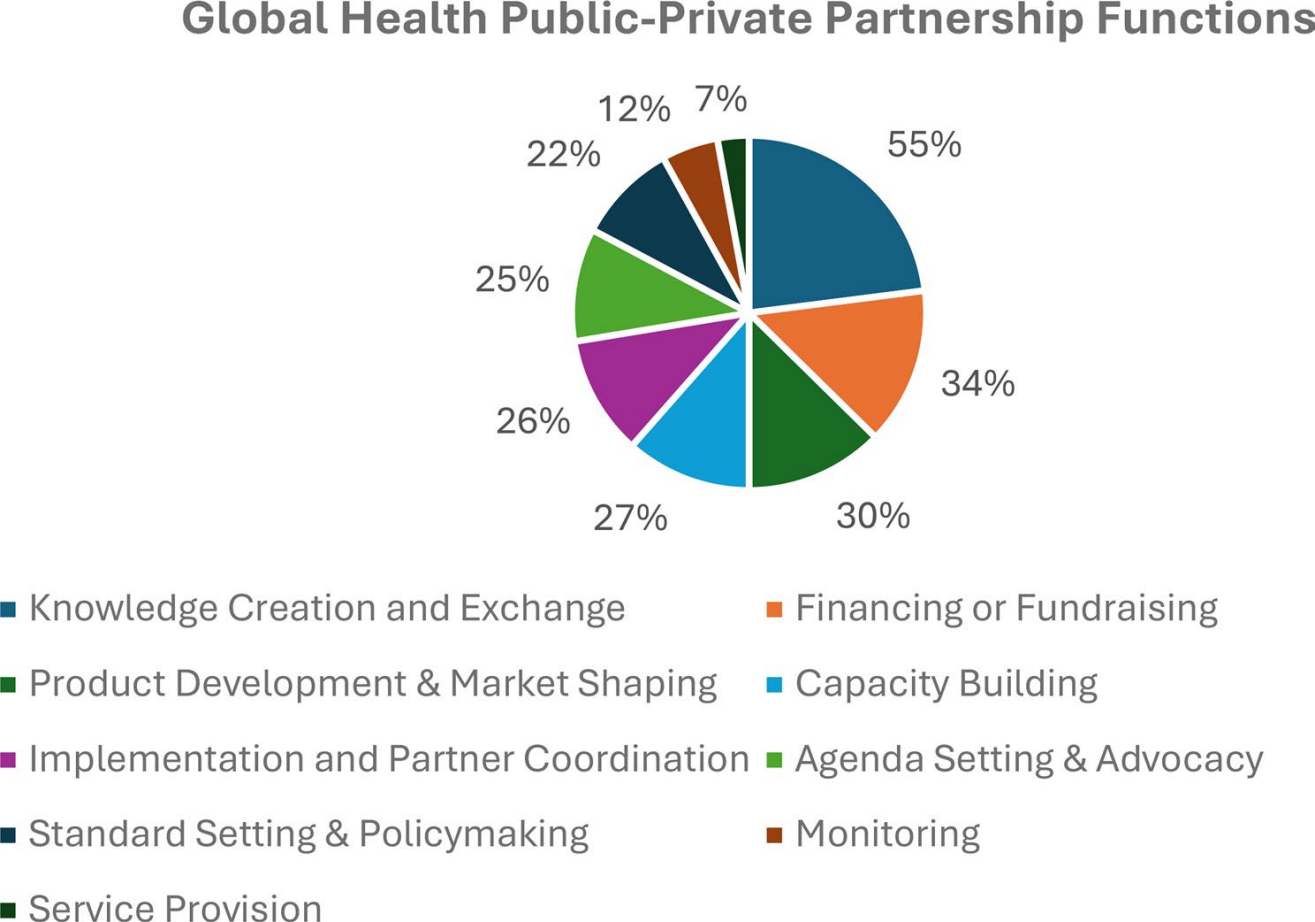
Functions of global health PPPs.^1^ Note that PPPs can have multiple functions and so the data in the figure do not add up to 73 PPPs or 100%

*Functions.* Figure 4 shows a well-distributed range of functions across the PPPs, the most common of which is knowledge creation and exchange (40/73, 55%) followed by global health financing or fundraising (25/73, 34%) and product development and market shaping (22/73, 30%). Least common is service provision (5/73, 7%), indicating that global health PPPs do not play a prominent role in country-level delivery of health services.

### Dataset constituency analysis

Descriptive analysis of constituency representation across the 73 global health PPPs in the dataset reveals striking disparities in access to governing venues by constituency type and country income-level. Table 1 summarizes the number and proportion of total seats per constituency, based if applicable, on the income-level of the country where that constituency is located. Enjoying the largest representation are high-income states (where state refers to government representatives of countries), which hold 170 out of 1,001 available seats, amounting to 17% of total seats across the 73 PPPs in the dataset. This is more than all seats held by low (18, 1.8%), lower-middle (55, 5.5%), and upper-middle (43, 4.3%) states combined. International organizations have the second highest representation across the PPPs with 123 seats, or 12.3% of all seats. Somewhat unexpectedly, the third most represented constituency are academic institutions from high-income countries (99, 9.9%), followed more predictably by the presence of companies (92, 9.2%), civil society organizations (80, 8%), independent representatives (57, 5.7%), and philanthropic foundations (40, 4%) from high-income countries. Besides PPPs (43, 4.3%), industry associations (24, 2.4%), and medical institutions (13, 1.3%) and community representatives (5, 0.5%) from high-income countries, the remaining 139 seats (13.9% of total seats) are split among academic institutions, civil society organizations, community, companies, independent representatives, philanthropic foundations, and medical institutions from low, lower-middle, or upper-middle income countries.

The righthand column of Table 1 frames these disparities in a slightly different way, highlighting the variation in average proportion of seats per PPP held by each constituency at each income level. It shows that, on average, high-income states hold 15.3% of seats per global health PPP, in contrast to 1.5% of seats for low-income states, and 4.7% and 3.2% of seats for lower-middle and upper-middle income states, respectively. These disparities by income hold for academic institutions, civil society organizations, companies, and independent representatives. For example, on average, academic institutions and companies in high-income countries each hold 10% of governing seats per PPP, compared to their counterparts in low-income countries, which hold an average of 0.7% and no seats, respectively, across those same PPPs.

**Table 1:** Distribution of global health PPP seats by constituency and income

Constituency	Country Income Level	No. Seats	Proportion of Total Seats (%)	Average Proportion of Seats per PPP (%)
Academic Institution	Low	5	0.5	0.7
	Lower-middle	22	2.2	2
	Upper-middle	12	1.2	1.5
	High	99	9.9	10.3
	Total	138	13.8	14.5
Civil Society Organization	Low	3	0.3	0.2
	Lower-middle	19	1.9	1.5
	Upper-middle	12	1.2	1.3
	High	80	8	8.7
	Total	114	11.4	11.7
Community	Low	1	0.1	0.1
	Lower-middle	8	0.8	0.5
	Upper-middle	5	0.5	0.3
	High	5	0.5	0.4
	Total	19	1.9	1.3
Company	Low	0	0	0
	Lower-middle	14	1.4	1.4
	Upper-middle	6	0.6	0.4
	High	92	9.2	10
	Total	112	11.2	11.8
Independent	Low	1	0.1	0.1
	Lower-middle	10	1	1.1
	Upper-middle	6	0.6	0.5
	High	57	5.7	6.7
	Total	74	7.4	8.4
Industry Association		24	2.4	2
International Organization		123	12.3	13.4
Medical Institution	Low	0	0	0
	Lower-middle	2	0.2	0.2
	Upper-middle	6	0.6	0.6
	High	13	1.3	1.1
	Total	21	2.1	1.9
Philanthropic Foundation	Low	0	0	0
	Lower-middle	3	0.3	0.4
	Upper-middle	4	0.4	0.5
	High	40	4.0	4.6
	Total	47	4.7	5.5
Public-Private Partnership		43	4.3	5.1
State	Low	18	1.8	1.5
	Lower-middle	55	5.5	4.7
	Upper-middle	43	4.3	3.2
	High	170	17	15.3
	Total	286	28.6	24.7

The constituency and income-based disparities in representation are perhaps more striking when considered in the aggregate, as presented in Tables 2 and 3. Grouped by constituency, states enjoy the most representation, both directly (286, 29%) and via international organizations, which hold the third greatest number of seats (123, 12%). Academic institutions are the second-most seat holders, with 138 seats and 14% of total seats when grouped by constituency. When combined across income, civil society organizations hold 11% of total seats and for-profit entities (companies and industry association) hold 13% of total seats. Least represented are community constituencies, who collectively hold just 19 seats, or 2% of total seats among global health PPPs. This is rather striking for a field that is forthright in its normative commitment to community engagement [39]. Table 3 shows that regional representation is concentrated in Europe and the Americas, which hold 34% and 27% of total available seats, respectively. When assessed in terms of average representation, this means that constituencies from Europe and the Americas (of which most are from the United States and Canada), typically take up 34% and 30% of seats on global health PPP governing boards. The remaining seats are split, on average, between representatives from Africa (14%), the Eastern Mediterranean (3%), Southeast Asia (4%), and the Western Pacific (8%), with the remaining 9% held by globally representative stakeholders (i.e., international organizations).

Table 4 reveals even more lopsidedness in representation, by categorizing the proportion of seats across global health PPPs by income. Grouped this way reveals that high-income country constituencies amass 69% of total seats across global health PPPs while low-income country constituencies have just 3% of total seats. Lower-middle and upper-middle income country constituencies split the remaining 28%, holding 16% and 12% of seats, respectively. While the direction of disparity is not unexpected, especially in light of other work in recent years on gender and geographic disparity in global health organization boards [14], the extent of inequitable access to decision-making in global health PPPs, which, by-and-large, target interventions in the Global South, is significant.

**Table 2:** Distribution of global health PPPs seats by constituency

Constituency	No. Seats	Proportion of Total Seats (%)
Academic Institution	138	14
Civil Society Organization	114	11
Community	19	2
Company	112	11
Independent	74	7
Industry Association	24	2
International Organization	123	12
Medical Institution	21	2
Philanthropic Foundation	47	5
Public-Private Partnership	43	4
State	286	29

**Table 3:** Distribution of global health PPPs seats by region

Region	Proportion of Total Seats (%)	Average Proportion of Seats per PPP (%)
Africa	14	14
Americas	27	30
Eastern Mediterranean	3	3
Europe	34	34
Southeast Asia	5	4
Western Pacific	9	8
Global	7	9

**Table 4:** Distribution of global health PPPs seats by income

Income Level	No. Seats	Proportion of Total Seats (%)
Low	28	3
Lower-Middle	133	16
Upper-Middle	94	12
High	556	69

^1^ These data exclude industry associations, international organizations, and PPPs

When broken down by actor, these disparities become even clearer. Table 5 presents the 30 actors with the most memberships across the global health PPPs; a full list is available in additional file 3. Of the 630 actors governing the 73 global health PPPs in the dataset, the WHO, United States government, and Gates Foundation hold governing seats in the greatest number of PPPs. The WHO leads with a governing seat in 21 PPPs, meaning that it is a governor in 29% of the PPPs in the dataset. The United States and Gates Foundation follow closely, with governing seats in 27% [20] and 26% [19] of PPPs, respectively. In terms of upper-middle income countries, Brazil (7, 10%), Indonesia (6, 8%), South Africa (6, 8%), and China (4, 6%) have some representation across the PPPs. India (9, 12%), Kenya (7, 10%), and Nigeria (6, 8%) are the only low or lower-middle income countries among these top represented actors, though the African Union and African Leaders Malaria Alliance as Africa region representatives hold governing roles in 6 (8% of) and 4 (6% of) PPPs, respectively. Interestingly, these data also show that two existing PPPs, The Global Fund (5, 7%) and Gavi (4, 6%), and an academic institution, the London School of Hygiene & Tropical Medicine (4, 6%), enjoy some of the top representation in governing roles across global health PPPs. Of these 30 actors, thirteen are Global North states (including the European Union).

**Table 5:** Actors with most seats in governing roles across the global health PPP dataset

Governing Memberships per Actor		
Actor	No. PPPs	Proportion of PPPs that Actor is a Governor (%)
World Health Organization	21	29
United States	20	27
Gates Foundation	19	26
United Kingdom	16	22
UNICEF	14	19
World Bank	14	19
Canada	12	16
Germany	12	16
Japan	12	16
European Union	11	15
France	10	14
India	9	12
Brazil	7	10
Kenya	7	10
Norway	7	10
African Union	6	8
Indonesia	6	8
Netherlands	6	8
Nigeria	6	8
South Africa	6	8
The Global Fund	5	7
South Korea	5	7
African Leaders Malaria Alliance	4	6
China	4	6
Gavi	4	6
Italy	4	6
London School of Hygiene & Tropical Medicine	4	6
Sweden	4	6
Switzerland	4	6
Wellcome Trust	4	6

Most actors have limited representation across the entire universe of PPPs. Indeed, a total of 526 actors have a governing role in only one PPP. Another 53 and 18 actors have two and three governing memberships, respectively, across the 73 PPPs. This includes – to contrast with the ample representation of high-income states in governing roles across the PPPs, as shown in Table 5 – most low or lower-middle income states. For example, while Canada and Germany have governing roles in 16% of PPPs, Bangladesh, Egypt, Pakistan, Tanzania, Uganda, and Zambia have governing seats in just 3% of PPPs (or two PPPs each). Low or lower-middle income states like Angola, Burkina Faso, Cambodia, Cameroon, El Salvador, Malawi, and Viet Nam have governing roles in just 1% of PPPs. Several high or upper-middle income non-state actors, in addition to those listed in Table 5, enjoy more representation in governing roles than these states, including Emory University, Goodbye Malaria (philanthropic foundation), Johnson & Johnson, Save the Children, and SwissTPH.

## Discussion

The analyses above support several observations about PPPs in global health governance. A central takeaway of these analyses, which I expand on at length below, is that global health PPPs appear to have allowed high-income country state and non-state actors to consolidate decision-making power over the health issues addressed by PPPs. Thus, while global health PPPs are inclusive insofar as they broaden the range of non-state actors that can participate in global health governance, they do not meaningfully include state and non-state governors from LMICs. Next, I offer a typology that differentiates global health PPPs based on their governing body composition. This typology joins existing efforts to define and categorize global health PPPs, whether by funding direction [27], nature of the partners’ relationship in terms of complexity, engagement, and reciprocity [40], degree of shared decision-making [27, 41], type of industry actors engaged [41], and policy stage and focus [41]. Finally, my dataset lends big picture support to recent small-n case study research on the rising PPP agency and authority in global health through inter-partnership cooperation [7]. Indeed, I find that PPPs have 43 governing seats across the 73 global health PPPs in the dataset and that two PPPs (Gavi and The Global Fund) are among the individual actors with the most representation across PPP governing bodies.

Returning to the core contribution of this study: who governs global health PPPs? One answer is that a striking 630 actors representing every region, income-level, and 109 countries govern 73 global health PPPs. And yet, decision-making influence, assessed based on the number and proportion of seats per actor and constituency, is not evenly distributed among these actors. A more accurate answer to this question would consider the breadth (i.e., how many PPPs an actor or constituency has a governing seat on) and depth (i.e., how many seats an actor or constituency has, on average, on PPP governing boards) of influence that actors and constituencies have across and within this universe of global health PPPs. The constituency analyses provide insights to this end. They show, unequivocally, that high-income countries, when considered in the aggregate across all constituency types, exercise the greatest influence in governance of global health PPPs, with 69% of total governing seats. In terms of breadth, the Global Oxygen Alliance, a super PPP governed by Africa CDC, PAHO, the Global Fund, and Unitaid, is the only PPP that does not have a high-income country governing representative. Though, eighteen high-income country stakeholders have indirect influence in the Alliance via the Global Fund and Unitaid boards. Regarding depth of decision-making influence, on average, high-income country representatives hold 57% of seats in PPP governing venues. In other words, high-income countries have, on average, majority decision-making power in global health PPPs.

When broken down by individual constituency, states retain the most influence by far; they hold 29% of all seats and on average, they have 25% of governing seats per PPP. When combined with international organizations (12% of total seats, average 13% of seats per PPP), the public sector holds 41% of total seats in global health PPPs, with an average of 38% of seats per PPP. This finding is consistent with other research showing that public sector organizations are the main partners in environmental PPPs [42]. Nonetheless, the fact that private sector actors cumulatively have majority representation in global health PPPs lends credence to concerns around the democratic deficit of PPPs [43, 44], and by extension, global health governance, given that global health PPPs exist in large numbers, have rising agency and authority [7], and attract substantial global health funding [7, 45]. These concerns are amplified when considering that of all seats held by states, 59% of them are occupied by high-income states. This translates to low, lower-middle, and upper-middle states holding just 2%, 6%, and 4% of total PPP seats, respectively, as compared to 17% of total PPP seats held by high-income states. Considered in terms of depth of influence, low, lower-middle, and upper-middle income states have just 2%, 5%, and 3%, on average, of seats per PPPs while high-income states hold, on average, 15% of seats per PPP. When assessed by actor, most low and middle-income countries have governing membership in just 1–2 global health PPPs (and of course, the countries not included in the dataset have zero direct representation). In contrast, six out of the 10 actors with the most governing memberships are high-income states.

For perspective, in the WHO – the primary multilateral venue for global health governance – the model of one country, one vote means that, at least in theory, low and middle-income countries have majority decision-making influence. Of course, in practice, major donors do exercise influence over the WHO’s agenda through earmarked funds [46, 47] and the perpetual underfunding of the WHO through assessed contributions stifles the organization’s progress on the programs of work voted in by member states [48]. The WHO’s inaugural turn to investment round style financing in November 2024, a fundraising strategy typically used by global health PPPs [49], may further dilute the quality of decision-making influence of non-donor countries in the organization if funds donated through replenishment events are earmarked for diseases donors are interested in, or if the WHO plans its investment case around those donor interests. However, this trend of donor-dominant influence plays out in PPPs as well [11, 50, 51]. Thus, based solely on representation in governing bodies, these analyses suggest that, in general, in global health PPPs, low and middle-income countries seem to have *less* decision-making influence than they do in the multilateral venue of the WHO, while high-income countries appear to enjoy *more* decision-making influence in global health PPPs as compared to the WHO.

Turning now to private sector constituencies, the community constituency is the only one where high-income country representatives do not vastly outnumber their low, lower-middle, and upper-middle income country counterparts. Indeed, for all other private sector constituencies (academic institution, civil society organization, company, independent, medical institution, and philanthropic foundation) high-income country representatives have more seats than all their low, lower-middle, and upper-middle income country counterparts combined. The prominent presence of academic institutions, which hold the second-highest proportion of seats (14%) across global health PPPs, is somewhat surprising given that they are typically not highlighted in analyses of PPP governance. Future research should explore why academic institutions engage in PPPs, including considering whether they are viewed and used as a strategy to secure funding.^4^ Furthermore, and reflecting the role of philanthropic foundations as brokers between the public and private sector in global health [52], the Gates Foundation has a governing role in a quarter (26%) of all global health PPPs. This breadth of influence held by a single actor is outmatched only by the WHO (29%) and the United States (27%). It may reflect the Gates Foundation’s participation in network diplomacy [53], such that it expands its relationships with states through co-governing PPPs. Lastly, when companies and industry associations are combined, they hold 14% of PPP governing seats, tying academic institutions for the second-highest proportion of seats. This means that for-profit constituencies have the second-highest representation in PPP governing boards. Still, this proportion of for-profit seats is much less than perhaps would be expected of PPPs, which are justified in part because they bring in the efficiency of the private sector [20, 34]. In all, these findings illustrate the value of breaking the private sector into more specific constituencies that reflect the wide variety of non-state actors that participate in global governance [54] – in lieu of the typical approach of discussing these constituents simply as businesses or non-governmental organizations.

The dataset and analyses have some limitations to consider. First, this research is premised on the idea that representation on a governing body is an indicator of decision-making power within the global health PPP. While this is a fair assumption, descriptive representation does not tell the full story of decision-making power and processes in global health PPPs. Representation alone, after all, does not necessarily translate into substantive influence, facilitate diverse perspectives and deliberation in PPPs, or consider board alliances [55–58]. Nonetheless, descriptive representation is a valuable shorthand for gaining a big picture understanding of the distribution of actor and constituency decision-making power within and across global health PPPs. Second, while PPP secretariats can exert some decision-making influence [7], this dataset cannot (and was not designed to) account for their influence because it is premised on principal-agent theory, where non-secretariat PPP board members, consistent with practice, have ultimately authority over the organization’s strategic, financial, and legal matters [22]. Future studies may consider ways to use large-n research to explore PPP secretariat decision-making influence. Third, because this dataset focuses on PPPs with governing boards, it by default excludes contract-based or programmatic PPPs without hybrid governance. This analytical scope is consistent with existing approaches to building PPP datasets and reflects the study’s interest in engaging debates on executive multistakeholderism [2] in global health governance. Two other limitations pertain to definitional decisions. Here, I recognize that my inclusion criterion requiring health to be an explicit goal of a PPP contradicts, to some extent, the social determinants of health framework central to global health practice, wherein factors outside the health sector determine health outcomes. While this choice was made to retain focus on PPPs with the most straightforward connection to global health governance, I recognize that there are many PPPs undertaking work relevant to social determinants of health that were not included in this dataset. Second, my definition of super PPP requires a threshold of at least 20% existing PPP representation on the PPP’s governing boards, so to indicate a blurring of direct influence via multiple layers of governance in line with Storeng et al., [28] original observation of this PPP type. Other scholars may suggest a higher threshold (e.g., 50%). For the time being, I see the 20% threshold as sufficient to distinguish super PPPs from other PPP types. Lastly, as PPP boards typically change every few years, it is important to see this dataset as a snapshot of board representation at one point in time.

## Conclusion

This dataset brings, to my knowledge, the most comprehensive, publicly accessible, and accurate information on PPPs active in contemporary global health governance. The descriptive analyses presented are an introduction to the dataset, which I hope other scholars and practitioners will build on to expand large-n analyses of global health PPPs. For now, this initial set of analyses find vast disparities in access to decision-making power within and across global health PPP governing bodies, and an overwhelming number of PPPs headquartered in Europe and North America. These trends are in direct contradiction to the growing normative demand to decolonize global health, calling into further question the legitimacy of these governing arrangements and contributing to a debate about the appropriate role and composition of PPPs in global health governance.

## Supplementary Information

Below is the link to the electronic supplementary material.

Supplementary Material 1

Supplementary Material 2

Supplementary Material 3

## Data Availability

A full list of the global health public-private partnerships and their corresponding typologies, and a list of all board members of these partnerships are available in additional files 1 and 2, respectively. Data on the headquarters of included partnerships is available in additional file 3. The datasets supporting the conclusions of this article, including the data presented in Tables 1–5, are available on the Harvard Dataverse: 10.7910/DVN/8MKNMJ.

## Footnotes

1 Thank you to a peer reviewer for this excellent point.

2 It is yet to be seen how the Trump Administration’s funding cuts and foreign policy will affect the United States’ representation in global health PPPs. For example, while the United States cut funding to Gavi, it still has representation on the board. This dataset offers a baseline from which to assess any changes to governing board membership when the dataset is updated in the future.

3 Public sector actors are those which are government or government-based entities. In global governance, this includes states and state-based international organizations.

4 Thank you to a reviewer who made this excellent point.

## Abbreviations

ACT-A: Access to COVID-19 Tools Accelerator
LMIC: Low and middle-income country
PPP: Public-private partnership
WHO: World Health Organization
