# Supplementary material for: The landscape of public-private partnerships in global health governance: introducing a new dataset
Source: Global Health. 2025 Nov 24;22:1. doi: 10.1186/s12992-025-01162-z (PMC12764153; doi:10.1186/s12992-025-01162-z)
Supplement: Supplementary file 1 — Supplementary Material 1 [file 12992_2025_1162_MOESM1_ESM.docx]

**Additional File 1**

**List of Public-Private Partnerships by Type**

The table below lists all public-private partnerships (PPPs) included in the dataset, categorized by the type of partnership they are according to the typology presented in the manuscript.

| **No.** | **Business PPPs** | **Civil Society PPPs** | **Trio PPPs** | **Super PPPs** |
| --- | --- | --- | --- | --- |
| 1 | Innovative Health Initiative | Alliance for Health Policy and Systems Research | AI4NTD | Access to COVID-19 Tools Accelerator |
| 2 | International Conference on Harmonization of Technical Requirements for Registration of Pharmaceuticals for Human Use | ATScale | Aspen Management Partnership for Health | Food Fortification Initiative |
| 3 |  | Drugs for Neglected Diseases initiative | Coalition for Epidemic Preparedness Innovations | Global Alliance for Vitamin A |
| 4 |  | European Observatory on Health Systems and Policies | Concept Foundation | Global Oxygen Alliance |
| 5 |  | Global Agriculture & Food Security Program | Family Planning 2030 | Global Water Operators' Partnership Alliance |
| 6 |  | Global Alliance to Eliminate Lymphatic Filariasis | Foundation for Innovative New Diagnostics |  |
| 7 |  | Global Antibiotic Research & Development Partnership | GAVI, The Vaccine Alliance |  |
| 8 |  | Global Financing Facility | Global Agenda for Sustainable Livestock |  |
| 9 |  | Global Outbreak Alert and Response Network | Global Alliance for Improved Nutrition |  |
| 10 |  | Global Partnership on Plastic Pollution and Marine Litter | Global Fund to Fight AIDS, Tuberculosis, and Malaria |  |
| 11 |  | Global Polio Eradication Initiative | Global Handwashing Partnership |  |
| 12 |  | Global Road Safety Facility | Global Health Innovation Technology Fund |  |
| 13 |  | Guidelines International Network | Global Partnership for Zero Leprosy |  |
| 14 |  | Health Technology Assessment International | Global Road Safety Partnership |  |
| 15 |  | International Centre of Insect Physiology and Ecology | Global Water Partnership |  |
| 16 |  | International Network of Food Data Systems | Health AI |  |
| 17 |  | International Union Against Tuberculosis and Lung Disease | Innovative Vector Control Consortium |  |
| 18 |  | Mectizan Donation Program | International AIDS Economics Network |  |
| 19 |  | Soil-Transmitted Helminths Coalition | International Crops Research Institute for the Semi-Arid Tropics |  |
| 20 |  | The Breast Health Global Initiative | International Maize and Wheat Improvement Centre |  |
| 21 |  | Unitaid | International Vaccine Institute |  |
| 22 |  |  | IODINE Global Network |  |
| 23 |  |  | Medicines for Malaria Venture |  |
| 24 |  |  | Medicines Patent Pool |  |
| 25 |  |  | Nutrition International |  |
| 26 |  |  | Pandemic Fund |  |
| 27 |  |  | Partnership for Influenza Vaccine Introduction |  |
| 28 |  |  | Project Last Mile |  |
| 29 |  |  | Research4Life |  |
| 30 |  |  | Reproductive Health Supplies Coalition |  |
| 31 |  |  | Roll Back Malaria Partnership to End Malaria |  |
| 32 |  |  | Sanitation and Water for All |  |
| 33 |  |  | Scaling Up Nutrition |  |
| 34 |  |  | Stop TB Partnership |  |
| 35 |  |  | Strategic Approach to International Chemicals Management |  |
| 36 |  |  | TB Alliance |  |
| 37 |  |  | The Global Alliance for Women's Health |  |
| 38 |  |  | The Partnership for Maternal, Newborn & Child Health |  |
| 39 |  |  | The Pediatric Praziquantel Consortium |  |
| 40 |  |  | Transform Health |  |
| 41 |  |  | UHC 2030 |  |
| 42 |  |  | UN Road Safety Fund |  |
| 43 |  |  | Unite4TB |  |
| 44 |  |  | Water Aid |  |
| 45 |  |  | World Vegetable Centre |  |
