# Supplementary material for: The landscape of public-private partnerships in global health governance: introducing a new dataset
Source: Global Health. 2025 Nov 24;22:1. doi: 10.1186/s12992-025-01162-z (PMC12764153; doi:10.1186/s12992-025-01162-z)
Supplement: Supplementary file 2 — Supplementary Material 2 [file 12992_2025_1162_MOESM2_ESM.docx]

**Additional File 2**

**Public-Private Partnership Headquarters**

The tables below summarize the headquarter location of the public-private partnerships included in the dataset. The first table summarizes this by region and the second by country.

| **Headquarter of Public-Private Partnerships by Region**  **(1 partnership removed due to no headquarter data)** | | |
| --- | --- | --- |
| **Region** | **Number Headquartered** | **Proportion Headquartered** |
| Africa | 3 | 4% |
| Americas | 22 | 31% |
| Eastern Mediterranean | 0 | 0% |
| Europe | 43 | 60% |
| Southeast Asia | 1 | 1% |
| Western Pacific | 3 | 4% |

| **Headquarter of Public-Private Partnerships by Country**  **(1 partnership removed due to no headquarter data)** | | |
| --- | --- | --- |
| **Country** | **Number Headquartered** | **Proportion Headquartered** |
| Belgium | 1 | 1% |
| Canada | 4 | 6% |
| Denmark | 1 | 1% |
| France | 1 | 1% |
| Germany | 1 | 1% |
| Great Britain | 2 | 3% |
| India | 1 | 1% |
| Italy | 2 | 3% |
| Japan | 1 | 1% |
| Kenya | 2 | 3% |
| Mexico | 1 | 1% |
| Netherlands | 3 | 4% |
| Norway | 1 | 1% |
| South Africa | 1 | 1% |
| South Korea | 1 | 1% |
| Sweden | 1 | 1% |
| Switzerland | 30 | 42% |
| Taiwan | 1 | 1% |
| United States of America | 17 | 24% |
