# Supplementary material for: The landscape of public-private partnerships in global health governance: introducing a new dataset
Source: Global Health. 2025 Nov 24;22:1. doi: 10.1186/s12992-025-01162-z (PMC12764153; doi:10.1186/s12992-025-01162-z)
Supplement: Supplementary file 3 — Supplementary Material 3 [file 12992_2025_1162_MOESM3_ESM.docx]

**Additional File 3**

**Full List of Governing Actors on Public-Private Partnership Boards**

The table below lists all 630 actors that are a governing member in at least one of the public-private partnerships (PPPs) included in the dataset. It displays how many PPPs each actor is a governing member of and the proportion of all PPPs in which the actor is a governor.

| **Full List of Actors in the Global Health Public-Private Partnership Dataset** | | | |
| --- | --- | --- | --- |
| **No.** | **Actor Name** | **Number of PPPs** | **Proportion of PPPs that Actor is a Governor (%)** |
| 1 | World Health Organization | 21 | 28.8% |
| 2 | United States | 20 | 27.4% |
| 3 | BMGF | 19 | 26.0% |
| 4 | United Kingdom | 16 | 21.9% |
| 5 | UNICEF | 14 | 19.2% |
| 6 | World Bank | 14 | 19.2% |
| 7 | Canada | 12 | 16.4% |
| 8 | Germany | 12 | 16.4% |
| 9 | Japan | 12 | 16.4% |
| 10 | European Commission or Union | 11 | 15.1% |
| 11 | France | 10 | 13.7% |
| 12 | India | 9 | 12.3% |
| 13 | Brazil | 7 | 9.6% |
| 14 | Kenya | 7 | 9.6% |
| 15 | Norway | 7 | 9.6% |
| 16 | African Union | 6 | 8.2% |
| 17 | Indonesia | 6 | 8.2% |
| 18 | Netherlands | 6 | 8.2% |
| 19 | Nigeria | 6 | 8.2% |
| 20 | South Africa | 6 | 8.2% |
| 21 | Global Fund | 5 | 6.8% |
| 22 | Korea | 5 | 6.8% |
| 23 | African Leaders Malaria Alliance | 4 | 5.5% |
| 24 | China | 4 | 5.5% |
| 25 | Gavi | 4 | 5.5% |
| 26 | Italy | 4 | 5.5% |
| 27 | LSHTM | 4 | 5.5% |
| 28 | Sweden | 4 | 5.5% |
| 29 | Switzerland | 4 | 5.5% |
| 30 | Wellcome Trust | 4 | 5.5% |
| 31 | Argentina | 3 | 4.1% |
| 32 | Emory University | 3 | 4.1% |
| 33 | Ethiopia | 3 | 4.1% |
| 34 | Finland | 3 | 4.1% |
| 35 | Food and Agriculture Organization | 3 | 4.1% |
| 36 | GAIN | 3 | 4.1% |
| 37 | Goodbye Malaria | 3 | 4.1% |
| 38 | International Federation of the Red Cross and Red Crescent Societies | 3 | 4.1% |
| 39 | Johnson & Johnson | 3 | 4.1% |
| 40 | KEMRI | 3 | 4.1% |
| 41 | Mali | 3 | 4.1% |
| 42 | Merck | 3 | 4.1% |
| 43 | Morocco | 3 | 4.1% |
| 44 | Nutrition International | 3 | 4.1% |
| 45 | Philippines | 3 | 4.1% |
| 46 | Save the Children | 3 | 4.1% |
| 47 | Spain | 3 | 4.1% |
| 48 | SwissTPH | 3 | 4.1% |
| 49 | UNFPA | 3 | 4.1% |
| 50 | Amref Health Africa | 2 | 2.7% |
| 51 | Astellas Pharma | 2 | 2.7% |
| 52 | Australia | 2 | 2.7% |
| 53 | AVAC | 2 | 2.7% |
| 54 | Bangladesh | 2 | 2.7% |
| 55 | Bonnie McClafferty | 2 | 2.7% |
| 56 | Boston University | 2 | 2.7% |
| 57 | Chile | 2 | 2.7% |
| 58 | China Agricultural University | 2 | 2.7% |
| 59 | Cornell University | 2 | 2.7% |
| 60 | Costa Rica | 2 | 2.7% |
| 61 | Egypt | 2 | 2.7% |
| 62 | EngenderHealth | 2 | 2.7% |
| 63 | ETH Zurich | 2 | 2.7% |
| 64 | Ferring Pharmaceuticals | 2 | 2.7% |
| 65 | FIA Foundation for the Automobile and Society | 2 | 2.7% |
| 66 | FIND | 2 | 2.7% |
| 67 | Gordon Dougan | 2 | 2.7% |
| 68 | Guyana | 2 | 2.7% |
| 69 | Helen Clark | 2 | 2.7% |
| 70 | Inter-American Development Bank | 2 | 2.7% |
| 71 | Inter-Parliamentary Union | 2 | 2.7% |
| 72 | Ireland | 2 | 2.7% |
| 73 | JCI Center for Global Health Diplomacy | 2 | 2.7% |
| 74 | Jeanette Vega | 2 | 2.7% |
| 75 | Johns Hopkins University | 2 | 2.7% |
| 76 | KNCV Tuberculosis Foundation | 2 | 2.7% |
| 77 | Luxembourg | 2 | 2.7% |
| 78 | Lygature | 2 | 2.7% |
| 79 | MSSRF | 2 | 2.7% |
| 80 | Novartis | 2 | 2.7% |
| 81 | Pakistan | 2 | 2.7% |
| 82 | Pharmaceutical Research and Manufacturers of America (PhRMA) | 2 | 2.7% |
| 83 | Radboud University Medical Centre | 2 | 2.7% |
| 84 | Saudi Arabia | 2 | 2.7% |
| 85 | Swedish University of Agricultural Sciences | 2 | 2.7% |
| 86 | Taiwan | 2 | 2.7% |
| 87 | Tanzania | 2 | 2.7% |
| 88 | Total Energies Foundation | 2 | 2.7% |
| 89 | Uganda | 2 | 2.7% |
| 90 | UNECE | 2 | 2.7% |
| 91 | UNESCAP | 2 | 2.7% |
| 92 | Unitaid | 2 | 2.7% |
| 93 | United Arab Emirates | 2 | 2.7% |
| 94 | University of California, San Fransisco | 2 | 2.7% |
| 95 | University of Ghent | 2 | 2.7% |
| 96 | University of Liverpool | 2 | 2.7% |
| 97 | University of Pretoria | 2 | 2.7% |
| 98 | University of Rwanda | 2 | 2.7% |
| 99 | Viatris | 2 | 2.7% |
| 100 | West African Health Organization | 2 | 2.7% |
| 101 | World Economic Forum | 2 | 2.7% |
| 102 | WWF | 2 | 2.7% |
| 103 | Zambia | 2 | 2.7% |
| 104 | 4SD Foundation | 1 | 1.4% |
| 105 | Aamer Ikram | 1 | 1.4% |
| 106 | Accenture Development Partnerships | 1 | 1.4% |
| 107 | Adrian Hopkins | 1 | 1.4% |
| 108 | Advanced Pediatrics Centre in Chandigarh | 1 | 1.4% |
| 109 | Aerium Therapeutics | 1 | 1.4% |
| 110 | Africa Finance Corporation | 1 | 1.4% |
| 111 | Africa Health Budget Network | 1 | 1.4% |
| 112 | African Civil Society Network on Water and Sanitation | 1 | 1.4% |
| 113 | African Development Bank | 1 | 1.4% |
| 114 | African Field Epidemiology Network | 1 | 1.4% |
| 115 | African Philanthropy Forum | 1 | 1.4% |
| 116 | African Population and Health Research Center | 1 | 1.4% |
| 117 | African Water and Sanitation Association | 1 | 1.4% |
| 118 | African Women Agribusiness Network Afrika | 1 | 1.4% |
| 119 | Afro Global Alliance Ghana | 1 | 1.4% |
| 120 | Aga Khan University East Africa | 1 | 1.4% |
| 121 | AGRA | 1 | 1.4% |
| 122 | AGREA Agricultural Systems International | 1 | 1.4% |
| 123 | AGREA Agricultural Systems International Inc. | 1 | 1.4% |
| 124 | AgResearch | 1 | 1.4% |
| 125 | agri benchmark | 1 | 1.4% |
| 126 | Al Azhar University | 1 | 1.4% |
| 127 | Alliance for Water Stewardship | 1 | 1.4% |
| 128 | American College of Physicians | 1 | 1.4% |
| 129 | American University of Beirut | 1 | 1.4% |
| 130 | Ana Lucía Arellano | 1 | 1.4% |
| 131 | Anders Nordstrom | 1 | 1.4% |
| 132 | Anesvad Foundation | 1 | 1.4% |
| 133 | Angola | 1 | 1.4% |
| 134 | Ann Moen | 1 | 1.4% |
| 135 | Ann Single | 1 | 1.4% |
| 136 | Anne Schuchat | 1 | 1.4% |
| 137 | Anthra | 1 | 1.4% |
| 138 | Apollo Asset Management | 1 | 1.4% |
| 139 | Apollo Hospitals Enterprise Ltd | 1 | 1.4% |
| 140 | Applied Horticultural Research | 1 | 1.4% |
| 141 | Aqua Publica Europea | 1 | 1.4% |
| 142 | AquaFed | 1 | 1.4% |
| 143 | Archer-Daniels-Midland Company | 1 | 1.4% |
| 144 | Ardent Mills | 1 | 1.4% |
| 145 | Armenia | 1 | 1.4% |
| 146 | Asia Pacific Leaders Malaria Alliance | 1 | 1.4% |
| 147 | Asian Development Bank | 1 | 1.4% |
| 148 | Asociación de Entes Reguladores de Agua Potable y Saneamiento de las Américas | 1 | 1.4% |
| 149 | Asociación Nacional de Empresas de Agua y Saneamiento | 1 | 1.4% |
| 150 | AstraZeneca | 1 | 1.4% |
| 151 | Austria | 1 | 1.4% |
| 152 | Autoliv Group | 1 | 1.4% |
| 153 | Avenir Health | 1 | 1.4% |
| 154 | Awa Marie Coll Seck | 1 | 1.4% |
| 155 | Axiom | 1 | 1.4% |
| 156 | Ayoade (Yodi) Alakija | 1 | 1.4% |
| 157 | Belgium | 1 | 1.4% |
| 158 | Ben-Gurion University of the Negev | 1 | 1.4% |
| 159 | Bern University | 1 | 1.4% |
| 160 | Bernard Pecoul | 1 | 1.4% |
| 161 | Bharat Biotech | 1 | 1.4% |
| 162 | Bience Gawanas | 1 | 1.4% |
| 163 | Biotechnology Innovation Organization (BIO) | 1 | 1.4% |
| 164 | Bipartisan Policy Center | 1 | 1.4% |
| 165 | Bloomberg Philanthropies | 1 | 1.4% |
| 166 | Blue Circle Diabetes Foundation | 1 | 1.4% |
| 167 | Botswana | 1 | 1.4% |
| 168 | Bridges to Development | 1 | 1.4% |
| 169 | Bridgestone Tires | 1 | 1.4% |
| 170 | Brigham & Women’s Hospital | 1 | 1.4% |
| 171 | Brooch Associates | 1 | 1.4% |
| 172 | Burkina Faso | 1 | 1.4% |
| 173 | Bush Institute | 1 | 1.4% |
| 174 | Cambodia | 1 | 1.4% |
| 175 | Cameroon | 1 | 1.4% |
| 176 | Cargill | 1 | 1.4% |
| 177 | Cari-WOP | 1 | 1.4% |
| 178 | Carolina Tornesi MacKinnon | 1 | 1.4% |
| 179 | Caroline Liew | 1 | 1.4% |
| 180 | CBMM | 1 | 1.4% |
| 181 | Center for Global Development | 1 | 1.4% |
| 182 | Center for Strategic and International Studies | 1 | 1.4% |
| 183 | Central Africa Republic | 1 | 1.4% |
| 184 | Centre for Health Sciences Training, Research and Development | 1 | 1.4% |
| 185 | CEPI | 1 | 1.4% |
| 186 | CGIAR | 1 | 1.4% |
| 187 | CHAI | 1 | 1.4% |
| 188 | Chandra Shekhar Azad University of Agriculture & Technology | 1 | 1.4% |
| 189 | Charles Mackenzie | 1 | 1.4% |
| 190 | Charlotte Maxeke Medical Research Cluster | 1 | 1.4% |
| 191 | China Disabled Persons Federation | 1 | 1.4% |
| 192 | Chugai Pharmaceutical | 1 | 1.4% |
| 193 | CIRAD | 1 | 1.4% |
| 194 | Cisco | 1 | 1.4% |
| 195 | City Cancer Challenge | 1 | 1.4% |
| 196 | Clima Investments | 1 | 1.4% |
| 197 | Coca-Cola Corporations | 1 | 1.4% |
| 198 | Coca-Cola Foundation | 1 | 1.4% |
| 199 | Columbia University | 1 | 1.4% |
| 200 | Community Initiative for Tuberculosis, HIV/AIDS and Malaria | 1 | 1.4% |
| 201 | Congolese Foundation for Medical Research | 1 | 1.4% |
| 202 | CORAF | 1 | 1.4% |
| 203 | Cote d'Ivoire | 1 | 1.4% |
| 204 | Critical Path Institute | 1 | 1.4% |
| 205 | CSL Vifor Pharma | 1 | 1.4% |
| 206 | Cyrus Ardalan | 1 | 1.4% |
| 207 | Daiichi Sankyo Company | 1 | 1.4% |
| 208 | Dana Hovig | 1 | 1.4% |
| 209 | David Norton | 1 | 1.4% |
| 210 | Deena Shiff | 1 | 1.4% |
| 211 | Delft Institute for Water Education | 1 | 1.4% |
| 212 | Deloitte Consulting, LL | 1 | 1.4% |
| 213 | Democratic Republic of Congo | 1 | 1.4% |
| 214 | Desmond FitzGerald | 1 | 1.4% |
| 215 | Development Bank of Latin America and the Caribbean | 1 | 1.4% |
| 216 | Dominican Republic | 1 | 1.4% |
| 217 | Dominique Limet | 1 | 1.4% |
| 218 | Dynatrace | 1 | 1.4% |
| 219 | Dzingai Mutumbuka | 1 | 1.4% |
| 220 | Early Childhood Development Action Network | 1 | 1.4% |
| 221 | East African Community | 1 | 1.4% |
| 222 | Eastern Mediterranean Public Health Network | 1 | 1.4% |
| 223 | EAT Foundation | 1 | 1.4% |
| 224 | eBASE Africa | 1 | 1.4% |
| 225 | Eisai Co | 1 | 1.4% |
| 226 | El Salvador | 1 | 1.4% |
| 227 | Elsevier | 1 | 1.4% |
| 228 | Enviro-Gro Farms Limited | 1 | 1.4% |
| 229 | Eswatini | 1 | 1.4% |
| 230 | Ethiopian Public Health Institute | 1 | 1.4% |
| 231 | Etteplan Oyj | 1 | 1.4% |
| 232 | Eurasian Coalition on Health, Rights, Gender and Sexual Diversity | 1 | 1.4% |
| 233 | EuropaBio | 1 | 1.4% |
| 234 | European Coordination Committee of the Radiological, Electromedical and Healthcare IT Industry | 1 | 1.4% |
| 235 | European Federation of Pharmaceutical Industries and Associations | 1 | 1.4% |
| 236 | European Healthcare Fraud and Corruption Network | 1 | 1.4% |
| 237 | European Lung Foundation | 1 | 1.4% |
| 238 | European Medicines Agency, EU | 1 | 1.4% |
| 239 | European Respiratory Society | 1 | 1.4% |
| 240 | European Transport Safety Council | 1 | 1.4% |
| 241 | European University Institute | 1 | 1.4% |
| 242 | Fatoumata Nafo | 1 | 1.4% |
| 243 | Felehansen | 1 | 1.4% |
| 244 | FHI 360 | 1 | 1.4% |
| 245 | Foothold Advisors Limited | 1 | 1.4% |
| 246 | Fòs Feminista | 1 | 1.4% |
| 247 | Frédéric Vallat | 1 | 1.4% |
| 248 | French National Health Insurance Fund - CNAM | 1 | 1.4% |
| 249 | Freshwater Action Network Mexico | 1 | 1.4% |
| 250 | Fridtjof Nansens Institutt | 1 | 1.4% |
| 251 | Friends for International Tuberculosis Relief | 1 | 1.4% |
| 252 | Gabriel Jaramillo | 1 | 1.4% |
| 253 | Gambia | 1 | 1.4% |
| 254 | GBCHealth | 1 | 1.4% |
| 255 | Genentech Roche | 1 | 1.4% |
| 256 | Geneva Graduate Institute | 1 | 1.4% |
| 257 | Georgetown University | 1 | 1.4% |
| 258 | Georgia | 1 | 1.4% |
| 259 | GESAMP | 1 | 1.4% |
| 260 | Gettysburg College | 1 | 1.4% |
| 261 | Ghana | 1 | 1.4% |
| 262 | Gillian Leng | 1 | 1.4% |
| 263 | Global Dairy Platform | 1 | 1.4% |
| 264 | Global Financing Facility | 1 | 1.4% |
| 265 | Global Fund for Widows | 1 | 1.4% |
| 266 | Global Health Council | 1 | 1.4% |
| 267 | Global Water Partnership, West Africa | 1 | 1.4% |
| 268 | Greece | 1 | 1.4% |
| 269 | Greenstar Social Marketing | 1 | 1.4% |
| 270 | Grégory Bonnaud | 1 | 1.4% |
| 271 | Grupo Siembra | 1 | 1.4% |
| 272 | GSK | 1 | 1.4% |
| 273 | Gustavo Murgel | 1 | 1.4% |
| 274 | Harvard | 1 | 1.4% |
| 275 | Health Care Without Harm | 1 | 1.4% |
| 276 | Health Foundation | 1 | 1.4% |
| 277 | Health Preparedness Partners | 1 | 1.4% |
| 278 | HealthforAnimals | 1 | 1.4% |
| 279 | Healthy Caribbean Coalition | 1 | 1.4% |
| 280 | Heifer International | 1 | 1.4% |
| 281 | Helen Keller International | 1 | 1.4% |
| 282 | Helena Special Investments | 1 | 1.4% |
| 283 | Helmholtz Zentrum München | 1 | 1.4% |
| 284 | Hong Kong Jockey Club | 1 | 1.4% |
| 285 | Hope for Future Generations | 1 | 1.4% |
| 286 | Hungary | 1 | 1.4% |
| 287 | Iberoamerican Cochrane Centre | 1 | 1.4% |
| 288 | IFPMA | 1 | 1.4% |
| 289 | Imperial College London | 1 | 1.4% |
| 290 | Indonesia Water Supply Association | 1 | 1.4% |
| 291 | Information Training & Outreach Centre for Africa | 1 | 1.4% |
| 292 | Ingenierios Sin Fronteras | 1 | 1.4% |
| 293 | Institut de Recherche Pour le Developpement | 1 | 1.4% |
| 294 | Institut de Recherche pour le Développement | 1 | 1.4% |
| 295 | Institut Pasteur | 1 | 1.4% |
| 296 | Instituto de Saude Publica da Universidade de Porto | 1 | 1.4% |
| 297 | Integrated Social Development Center | 1 | 1.4% |
| 298 | Inter-Organization Programme for the Sound Management of Chemicals | 1 | 1.4% |
| 299 | International Association for Integration, Dignity and Economic Advancement | 1 | 1.4% |
| 300 | International Association of Operative Millers | 1 | 1.4% |
| 301 | International Association of Scientific, Technical & Medical Publishers | 1 | 1.4% |
| 302 | International Atomic Energy Agency | 1 | 1.4% |
| 303 | International Center for Research on Women | 1 | 1.4% |
| 304 | International Center of Excellence in Research | 1 | 1.4% |
| 305 | International Center on Agriculture Research in Drylands | 1 | 1.4% |
| 306 | International Centre for Diarrhoeal Disease Research, Bangladesh | 1 | 1.4% |
| 307 | International Centre for Health Systems Strengthening | 1 | 1.4% |
| 308 | International Confederation of Midwives | 1 | 1.4% |
| 309 | International Council of Chemical Associations | 1 | 1.4% |
| 310 | International Council of Nurses | 1 | 1.4% |
| 311 | International Dairy Federation | 1 | 1.4% |
| 312 | International Disability Alliance | 1 | 1.4% |
| 313 | International Federation for Spina Bifida and Hydrocephalus | 1 | 1.4% |
| 314 | International Federation of Anti-Leprosy Associations | 1 | 1.4% |
| 315 | International Federation of Gynecology and Obstetrics | 1 | 1.4% |
| 316 | International Feed Industry Federation | 1 | 1.4% |
| 317 | International Generic and Biosimilar Medicines Association (IGBA) | 1 | 1.4% |
| 318 | International Leprosy Association | 1 | 1.4% |
| 319 | International Livestock Research Institute | 1 | 1.4% |
| 320 | International Maritime Organization | 1 | 1.4% |
| 321 | International Meat Secretariat | 1 | 1.4% |
| 322 | International Pediatric Association | 1 | 1.4% |
| 323 | International Pollutants Elimination Network | 1 | 1.4% |
| 324 | International Poultry Council | 1 | 1.4% |
| 325 | International Road Victims’ Partnership | 1 | 1.4% |
| 326 | International Severe Acute Respiratory and Emerging Infection Consortium | 1 | 1.4% |
| 327 | International SOS Foundation | 1 | 1.4% |
| 328 | International Trade Union Confederation | 1 | 1.4% |
| 329 | International Union of Nutritional Sciences | 1 | 1.4% |
| 330 | International Water Management Institute | 1 | 1.4% |
| 331 | Ipas Latin America and the Caribbean | 1 | 1.4% |
| 332 | Iran | 1 | 1.4% |
| 333 | IRC International Water and Sanitation Centre | 1 | 1.4% |
| 334 | Islamic Development Bank | 1 | 1.4% |
| 335 | IXALTIS | 1 | 1.4% |
| 336 | J.P. Morgan Chase & Co. | 1 | 1.4% |
| 337 | Jaehyang So | 1 | 1.4% |
| 338 | Jakaya Mrisho Kikwete Foundation | 1 | 1.4% |
| 339 | Jamaica | 1 | 1.4% |
| 340 | Jan A. Delcour | 1 | 1.4% |
| 341 | Jane Halton | 1 | 1.4% |
| 342 | Japan Anti-TB Association | 1 | 1.4% |
| 343 | Japan Pharmaceutical Manufacturers Association | 1 | 1.4% |
| 344 | JBI Adelaide | 1 | 1.4% |
| 345 | Jennifer Blanke | 1 | 1.4% |
| 346 | Jeremy Lefroy | 1 | 1.4% |
| 347 | Jimma University | 1 | 1.4% |
| 348 | Jo Carol Hiat | 1 | 1.4% |
| 349 | John Vincent Fieno | 1 | 1.4% |
| 350 | Jon Lomøy | 1 | 1.4% |
| 351 | Jon Schofield | 1 | 1.4% |
| 352 | José Manuel Barroso | 1 | 1.4% |
| 353 | JPC5 Consulting | 1 | 1.4% |
| 354 | Julia Bunting | 1 | 1.4% |
| 355 | Julie Wynne | 1 | 1.4% |
| 356 | Kapa D. Ramaiah | 1 | 1.4% |
| 357 | Karen Sørensen | 1 | 1.4% |
| 358 | Kasetsart University | 1 | 1.4% |
| 359 | Kedaara Capital | 1 | 1.4% |
| 360 | Khon Kaen University | 1 | 1.4% |
| 361 | Kingdom of Bahrain | 1 | 1.4% |
| 362 | Kiwanis International | 1 | 1.4% |
| 363 | Koei Chemical | 1 | 1.4% |
| 364 | Kwame Akuffo-Akoto | 1 | 1.4% |
| 365 | Kyrgyz Republic | 1 | 1.4% |
| 366 | Laboratorios Liomont | 1 | 1.4% |
| 367 | LAC Key Correspondent Team | 1 | 1.4% |
| 368 | Lagata | 1 | 1.4% |
| 369 | Lancaster University | 1 | 1.4% |
| 370 | Latinamerican and Caribbean Society of Medical Oncology | 1 | 1.4% |
| 371 | Laurence Amand Jules | 1 | 1.4% |
| 372 | Leading Light Initiative | 1 | 1.4% |
| 373 | League for Pastoral Peoples and Endogenous Livestock Development | 1 | 1.4% |
| 374 | Leibniz Lung Centre | 1 | 1.4% |
| 375 | Leprosy Research Initiative | 1 | 1.4% |
| 376 | Lexicon Advisory | 1 | 1.4% |
| 377 | LGT Venture Philanthropy | 1 | 1.4% |
| 378 | LMU University Hospital Munich | 1 | 1.4% |
| 379 | London School of Hygiene & Tropical Medicine | 1 | 1.4% |
| 380 | Louis Da Gama | 1 | 1.4% |
| 381 | LSE | 1 | 1.4% |
| 382 | Lucille H Blumberg | 1 | 1.4% |
| 383 | Lymphatic Filariasis Non-Governmental Development Organization Network | 1 | 1.4% |
| 384 | M.M. Dillon & Co. | 1 | 1.4% |
| 385 | Malawi | 1 | 1.4% |
| 386 | Malaysia | 1 | 1.4% |
| 387 | Mamotest | 1 | 1.4% |
| 388 | Management Sciences for Health | 1 | 1.4% |
| 389 | Marc Hofstetter | 1 | 1.4% |
| 390 | Marie-Paule Kien | 1 | 1.4% |
| 391 | Marie-Paule Kieny | 1 | 1.4% |
| 392 | Marisol Touraine | 1 | 1.4% |
| 393 | Mastercard | 1 | 1.4% |
| 394 | McMaster University | 1 | 1.4% |
| 395 | Medecins Sans Frontiers International | 1 | 1.4% |
| 396 | Medic | 1 | 1.4% |
| 397 | Medicines for Malaria Venture | 1 | 1.4% |
| 398 | Medicus Mundi Switzerland | 1 | 1.4% |
| 399 | Mediterranean Action Plan | 1 | 1.4% |
| 400 | Medtronic Global Technology and Innovation | 1 | 1.4% |
| 401 | Meridian Institute | 1 | 1.4% |
| 402 | Mexican Association of the Salt Industry | 1 | 1.4% |
| 403 | Mexican Red Cross/Cruz Roja Mexicana | 1 | 1.4% |
| 404 | Mexico | 1 | 1.4% |
| 405 | Michèle Costafrolaz | 1 | 1.4% |
| 406 | Michelin Corporate Foundation | 1 | 1.4% |
| 407 | Michelin Group | 1 | 1.4% |
| 408 | Micronutrient Forum | 1 | 1.4% |
| 409 | Migrant Clinician Network | 1 | 1.4% |
| 410 | Moderna | 1 | 1.4% |
| 411 | Monash University | 1 | 1.4% |
| 412 | Mongolia | 1 | 1.4% |
| 413 | Montenegro | 1 | 1.4% |
| 414 | Movement for the Reintegration of Persons Affected by Hansen’s Disease (MORHAN) | 1 | 1.4% |
| 415 | Mphu Ramatlapeng | 1 | 1.4% |
| 416 | MSF | 1 | 1.4% |
| 417 | MSI Reproductive Choices | 1 | 1.4% |
| 418 | Muller Automotive | 1 | 1.4% |
| 419 | Mundo Sano Foundation | 1 | 1.4% |
| 420 | My Empowerment Pack | 1 | 1.4% |
| 421 | Naguib Kheraj | 1 | 1.4% |
| 422 | Nam Hee Kim | 1 | 1.4% |
| 423 | National Leprosy Elimination Programme of Ghana, Ghana Health Service | 1 | 1.4% |
| 424 | National Livestock Confederation | 1 | 1.4% |
| 425 | National Medical Products Administration (NMPA) | 1 | 1.4% |
| 426 | National Respiratory Referral Hospital Jakarta | 1 | 1.4% |
| 427 | National University of Salta | 1 | 1.4% |
| 428 | National University of Singapore | 1 | 1.4% |
| 429 | Nepal | 1 | 1.4% |
| 430 | Nestle | 1 | 1.4% |
| 431 | Nevin Scrimshaw International Nutrition Foundation | 1 | 1.4% |
| 432 | New Zealand | 1 | 1.4% |
| 433 | Next Gen Nutrition Investment Partners, LLC | 1 | 1.4% |
| 434 | Niteen Wairagkar | 1 | 1.4% |
| 435 | North Macedonia | 1 | 1.4% |
| 436 | Ocean Conservancy | 1 | 1.4% |
| 437 | One Health Trust | 1 | 1.4% |
| 438 | Oregon Health & Science University | 1 | 1.4% |
| 439 | Oswaldo Cruz Foundation/Fiocruz | 1 | 1.4% |
| 440 | Otsuka Novel Products GmbH | 1 | 1.4% |
| 441 | Ouagadougou Partnership | 1 | 1.4% |
| 442 | Oxford Policy Management | 1 | 1.4% |
| 443 | Oxford University | 1 | 1.4% |
| 444 | Pacific Community | 1 | 1.4% |
| 445 | Pacific Waste and Water Association | 1 | 1.4% |
| 446 | Pakistan Water Operators Network | 1 | 1.4% |
| 447 | Palestine | 1 | 1.4% |
| 448 | Pamela Steele Associates | 1 | 1.4% |
| 449 | Paraguay | 1 | 1.4% |
| 450 | Paul Herrling | 1 | 1.4% |
| 451 | Pentecostal Advocates for Socio-Economic Development | 1 | 1.4% |
| 452 | Pepsico | 1 | 1.4% |
| 453 | Persagabatan General Hospital | 1 | 1.4% |
| 454 | Pfizer | 1 | 1.4% |
| 455 | PharmaJet | 1 | 1.4% |
| 456 | Phyllis Heydt | 1 | 1.4% |
| 457 | Plan International | 1 | 1.4% |
| 458 | Poland | 1 | 1.4% |
| 459 | Population Council Zambia | 1 | 1.4% |
| 460 | Population Services International | 1 | 1.4% |
| 461 | Portugal | 1 | 1.4% |
| 462 | Praisegate Services & Consult | 1 | 1.4% |
| 463 | Prince Mohamed Bin Abdulaziz Hospital | 1 | 1.4% |
| 464 | Procter & Gamble | 1 | 1.4% |
| 465 | Profamilia | 1 | 1.4% |
| 466 | PT Bank Mandiri tbk | 1 | 1.4% |
| 467 | Public Citizen | 1 | 1.4% |
| 468 | Public Services International | 1 | 1.4% |
| 469 | Qatar | 1 | 1.4% |
| 470 | Qatar Energy | 1 | 1.4% |
| 471 | Queensland University of Technology | 1 | 1.4% |
| 472 | REACH India | 1 | 1.4% |
| 473 | Reaching the Last Mile | 1 | 1.4% |
| 474 | Research Center Borstel | 1 | 1.4% |
| 475 | RESULTS Educational Fund | 1 | 1.4% |
| 476 | RiboNova Inc. | 1 | 1.4% |
| 477 | Richard Warren Shepro | 1 | 1.4% |
| 478 | Rick Bright | 1 | 1.4% |
| 479 | Roche Diagnostics International Ltd | 1 | 1.4% |
| 480 | Roche Pharmaceuticals | 1 | 1.4% |
| 481 | Roslyn Morauta | 1 | 1.4% |
| 482 | Rotary International | 1 | 1.4% |
| 483 | Royal Dutch DSM | 1 | 1.4% |
| 484 | RTI International | 1 | 1.4% |
| 485 | Rumishael Shoo | 1 | 1.4% |
| 486 | Rwanda | 1 | 1.4% |
| 487 | S.C. Johnson & Sons, Inc | 1 | 1.4% |
| 488 | Sahel Consulting Agriculture & Nutrition | 1 | 1.4% |
| 489 | Sally Timpson | 1 | 1.4% |
| 490 | Santa Cruz IP | 1 | 1.4% |
| 491 | Sara Ryerson | 1 | 1.4% |
| 492 | Sasakawa Health Foundation | 1 | 1.4% |
| 493 | Sathguru Management Consultants | 1 | 1.4% |
| 494 | School of Public Health of Mexico | 1 | 1.4% |
| 495 | Sciences Po | 1 | 1.4% |
| 496 | Scottish Intercollegiate Guidelines Network | 1 | 1.4% |
| 497 | Senegal | 1 | 1.4% |
| 498 | Seychelles | 1 | 1.4% |
| 499 | Shell | 1 | 1.4% |
| 500 | Shionogi & Co | 1 | 1.4% |
| 501 | SickKids Centre for Global Child Health | 1 | 1.4% |
| 502 | Siemens Healthineers | 1 | 1.4% |
| 503 | Silke Lindenberg | 1 | 1.4% |
| 504 | Slovenia | 1 | 1.4% |
| 505 | Solomon Islands | 1 | 1.4% |
| 506 | Somalia | 1 | 1.4% |
| 507 | South-Eastern Europe Regional HIV and TB Community Network | 1 | 1.4% |
| 508 | Southern Africa Federation of the Disabled | 1 | 1.4% |
| 509 | Southern Africa Miners Association | 1 | 1.4% |
| 510 | Southern African Development Community | 1 | 1.4% |
| 511 | Speak Up Africa | 1 | 1.4% |
| 512 | St. John’s Medical College | 1 | 1.4% |
| 513 | St. Pölten University of Applied Sciences | 1 | 1.4% |
| 514 | Stalker Radar | 1 | 1.4% |
| 515 | Stanislas Zuin | 1 | 1.4% |
| 516 | Stockholm Environment Institute | 1 | 1.4% |
| 517 | Stop TB Partnership Kenya | 1 | 1.4% |
| 518 | Stop TB Partnership Nigeria | 1 | 1.4% |
| 519 | Strong Harbor, LLC | 1 | 1.4% |
| 520 | Success Capital | 1 | 1.4% |
| 521 | Susan G. Komen for the Cure | 1 | 1.4% |
| 522 | Susan Russell | 1 | 1.4% |
| 523 | Susan Thompson Buffet Foundation | 1 | 1.4% |
| 524 | Takeda Pharmaceutical Company Limited | 1 | 1.4% |
| 525 | TASK Clinical Research Centre | 1 | 1.4% |
| 526 | Tayf | 1 | 1.4% |
| 527 | TB Alliance | 1 | 1.4% |
| 528 | TBNet | 1 | 1.4% |
| 529 | TBpeople Ukraine | 1 | 1.4% |
| 530 | Technical University of Munich | 1 | 1.4% |
| 531 | Teresa Ressel | 1 | 1.4% |
| 532 | Texas A&M University | 1 | 1.4% |
| 533 | Thailand | 1 | 1.4% |
| 534 | The Association of NGOs (TANGO) | 1 | 1.4% |
| 535 | The Brazilian Leprosy Program / Giselda Trigueiro Hospital | 1 | 1.4% |
| 536 | The Carter Center | 1 | 1.4% |
| 537 | The Crawford Fund | 1 | 1.4% |
| 538 | The Institute for Global Health and Development of Guinea-Bissau | 1 | 1.4% |
| 539 | The International Initiative for Impact Evaluation (3ie) | 1 | 1.4% |
| 540 | The International Planned Parenthood Federation | 1 | 1.4% |
| 541 | The Leprosy Mission International | 1 | 1.4% |
| 542 | The PACT | 1 | 1.4% |
| 543 | The Rockefeller Foundation | 1 | 1.4% |
| 544 | The University of Queensland | 1 | 1.4% |
| 545 | Tiba Biotech | 1 | 1.4% |
| 546 | Togo | 1 | 1.4% |
| 547 | Toilet Board Coalition | 1 | 1.4% |
| 548 | Tony Blair Institute for Global Change | 1 | 1.4% |
| 549 | Transform Health | 1 | 1.4% |
| 550 | Tribe of Sustainability Change Agents | 1 | 1.4% |
| 551 | Tribhuvan University | 1 | 1.4% |
| 552 | Tunisia | 1 | 1.4% |
| 553 | Turkey | 1 | 1.4% |
| 554 | TWEET Foundation | 1 | 1.4% |
| 555 | UN Sanitation & Hygiene Fund | 1 | 1.4% |
| 556 | UN Secretary-General’s Special Envoy for Road Safety, United Nations | 1 | 1.4% |
| 557 | UNAIDS | 1 | 1.4% |
| 558 | UNECA | 1 | 1.4% |
| 559 | United Nations Environmental Programme | 1 | 1.4% |
| 560 | United Nations Foundation | 1 | 1.4% |
| 561 | United Nations University | 1 | 1.4% |
| 562 | Università Cattolica del Sacro Cuore | 1 | 1.4% |
| 563 | Université Cheikh Anta Diop de Dakar | 1 | 1.4% |
| 564 | University College London | 1 | 1.4% |
| 565 | University Felix Houphouet-Boigny | 1 | 1.4% |
| 566 | University in Owerri | 1 | 1.4% |
| 567 | University of Bergen | 1 | 1.4% |
| 568 | University of California, Berkley | 1 | 1.4% |
| 569 | University of Cambridge | 1 | 1.4% |
| 570 | University of Campinas | 1 | 1.4% |
| 571 | University of Capetown | 1 | 1.4% |
| 572 | University of Chile | 1 | 1.4% |
| 573 | University of Costa Rica | 1 | 1.4% |
| 574 | University of Dundee | 1 | 1.4% |
| 575 | University of Guyana | 1 | 1.4% |
| 576 | University of Hamburg | 1 | 1.4% |
| 577 | University of Lagos | 1 | 1.4% |
| 578 | University of Malawi | 1 | 1.4% |
| 579 | University of Milano | 1 | 1.4% |
| 580 | University of Montpellier | 1 | 1.4% |
| 581 | University of Nebraska | 1 | 1.4% |
| 582 | University of Neuchâtel | 1 | 1.4% |
| 583 | University of Ottawa | 1 | 1.4% |
| 584 | University of Pennsylvania | 1 | 1.4% |
| 585 | University of St. Andrews | 1 | 1.4% |
| 586 | University of Sydney | 1 | 1.4% |
| 587 | University of Texas Southwestern | 1 | 1.4% |
| 588 | University of Toronto | 1 | 1.4% |
| 589 | University of Washington | 1 | 1.4% |
| 590 | University of Waterloo | 1 | 1.4% |
| 591 | University of Wisconsin-Madison | 1 | 1.4% |
| 592 | University of Witwatersrand in South Africa | 1 | 1.4% |
| 593 | Unlimit Health | 1 | 1.4% |
| 594 | UNOPS | 1 | 1.4% |
| 595 | UNSW Sydney | 1 | 1.4% |
| 596 | UPDC Real Estate Investment Trust | 1 | 1.4% |
| 597 | Uppsala University | 1 | 1.4% |
| 598 | Uruguay | 1 | 1.4% |
| 599 | USA National Academy of Medicine | 1 | 1.4% |
| 600 | Utrecht University | 1 | 1.4% |
| 601 | Vatera Healthcare Partners | 1 | 1.4% |
| 602 | Vested World | 1 | 1.4% |
| 603 | Vestergaard | 1 | 1.4% |
| 604 | Vétérinaires Sans Frontières | 1 | 1.4% |
| 605 | Viet Nam | 1 | 1.4% |
| 606 | VILD Foundation | 1 | 1.4% |
| 607 | Village Reach | 1 | 1.4% |
| 608 | Vita-Salute San Raffaele University | 1 | 1.4% |
| 609 | VivaValet | 1 | 1.4% |
| 610 | Water Asset Management LLC | 1 | 1.4% |
| 611 | Water Integrity Network | 1 | 1.4% |
| 612 | WaterLinks | 1 | 1.4% |
| 613 | WEnergy Global Pte Ltd | 1 | 1.4% |
| 614 | WIPO | 1 | 1.4% |
| 615 | WOP-LAC | 1 | 1.4% |
| 616 | World Alliance of Mobile Indigenous Peoples, Argentina | 1 | 1.4% |
| 617 | World Alliance of Mobile Indigenous Peoples, North Africa | 1 | 1.4% |
| 618 | World Food Programme | 1 | 1.4% |
| 619 | World Hepatitis Alliance | 1 | 1.4% |
| 620 | World Organisation for Animal Health (OIE) | 1 | 1.4% |
| 621 | World Rescue Organization | 1 | 1.4% |
| 622 | Wote Youth Development Projects | 1 | 1.4% |
| 623 | Yibing Wu | 1 | 1.4% |
| 624 | Yilma Kebede | 1 | 1.4% |
| 625 | Youth Advocacy Network Sri Lanka | 1 | 1.4% |
| 626 | Yuli Ismartono | 1 | 1.4% |
| 627 | Yvonne Chaka Chaka | 1 | 1.4% |
| 628 | Zenith Water Projects | 1 | 1.4% |
| 629 | Zimbabwe | 1 | 1.4% |
| 630 | Zurich Insurance Group | 1 | 1.4% |
